# 5,6-Epoxycholesterol Isomers Induce Oxiapoptophagy in Myeloma Cells

**DOI:** 10.3390/cancers13153747

**Published:** 2021-07-26

**Authors:** Oumaima Jaouadi, Inès Limam, Mohamed Abdelkarim, Emna Berred, Ahlem Chahbi, Mélody Caillot, Brigitte Sola, Fatma Ben Aissa-Fennira

**Affiliations:** 1 Laboratory of Oncohematology, PRF of Oncohematology, Faculty of Medicine of Tunis, Tunis El Manar University, Tunis 1006, Tunisia; oumaima.jaouadi@fst.utm.tn (O.J.); ines.limam@fmt.utm.tn (I.L.); mohamed.abdelkarim@fmt.utm.tn (M.A.); emna.berred@fmt.utm.tn (E.B.); ahlem.chahbi@fmt.utm.tn (A.C.); 2 Department of Clinical Hematology, Aziza Othmana Hospital, Tunis 1006, Tunisia; 3 Normandie University, INSERM U1245, Unicaen, F-14000 Caen, France; melody.caillot@unicaen.fr (M.C.); brigitte.sola@unicaen.fr (B.S.)

**Keywords:** apoptosis, autophagy, anti-tumor activity, multiple myeloma, oxiapotophagy, oxidative stress, oxysterols, synergistic interaction

## Abstract

**Simple Summary:**

As the second most frequent hematological malignancy, multiple myeloma remains incurable with recurrent patient relapse due to drug resistance. Therefore, the development of novel and potent therapies is urgently required. Herein, we demonstrated the anti-tumor activity of 5,6 α- and 5,6 β-epoxycholesterol isomers against human myeloma cells. Our results highlighted a striking anti-myeloma efficiency of these bioactive molecules and their added value in future potential treatments including combination therapy of multiple myeloma.

**Abstract:**

Multiple myeloma (MM) is an incurable plasma cell malignancy with frequent patient relapse due to innate or acquired drug resistance. Cholesterol metabolism is reported to be altered in MM; therefore, we investigated the potential anti-myeloma activity of two cholesterol derivatives: the 5,6 α- and 5,6 β-epoxycholesterol (EC) isomers. To this end, viability assays were used, and isomers were shown to exhibit important anti-tumor activity in vitro in JJN3 and U266 human myeloma cell lines (HMCLs) and ex vivo in myeloma patients’ sorted CD138+ malignant cells. Moreover, we confirmed that 5,6 α-EC and 5,6 β-EC induced oxiapoptophagy through concomitant oxidative stress and caspase-3-mediated apoptosis and autophagy. Interestingly, in combination treatment a synergistic interaction was observed between 5,6 α-EC and 5,6 β-EC on myeloma cells. These data highlight a striking anti-tumor activity of 5,6 α-EC and 5,6 β-EC bioactive molecules against human myeloma cells, paving the way for their potential role in future therapeutic strategies in MM.

## 1. Introduction

Multiple myeloma (MM) is a relapsed/refractory malignant hematological disease characterized by a clonal proliferation of tumor plasma cells in the bone marrow and/or extra-medullary sites. It represents the second most common hematological malignancy [1]. Despite the development of novel therapeutic strategies, including proteasome inhibitors, drug resistance occurs with frequent disease relapse and fatal outcome [2]. Therefore, there is a crucial need to design more effective anti-myeloma therapies by discovering novel bioactive compounds and combination strategies [3,4].

The metabolism of cholesterol plays a role in tumor development, drug resistance, and drug pharmacology for various cancers including MM [5,6]. Among cholesterol metabolites, oxysterols represent a large family of lipids implicated in a plethora of physiological processes [5]. They are produced through enzymatic reactions or by auto-oxidation [7], and can also be provided by food [8]. Moreover, oxysterols have been shown to interfere with proliferation and cause many types of cell death including oxiapoptophagy in various cancers, such as glioblastoma, colon, breast, prostate cancers and hematologic malignancies [5,9]. Among them, the 5,6-epoxycholesterols (5,6-ECs), isomers α (5,6 α-EC) and β (5,6 β-EC), 27-carbon molecules, are enzymatically generated by cholesterol oxidation at the ∆5 double-bond between C5 and C6 of the B ring of the steroid backbone [10]. The cytotoxic effect of 5,6 β-EC against human U937 promonocytic leukemia cells has been described previously, whereas the corresponding α isomer had no such property [11]. Moreover, both isomers are able to enhance superoxide anions (O^2−^) production, suggesting a pro-oxidative activity. Interestingly, the 5,6-ECs possess both pro-tumor and anti-oncogenic properties the latter through the production of steroidal alkaloids [12]. Tamoxifen, widely used for breast cancer therapy, stimulates the production and accumulation of 5,6-ECs in a reactive oxygen species (ROS)-dependent manner [12]. Moreover, in MM, previous data reported that altering cholesterol metabolism is a means to induce cell death. Indeed, the 4-hydroxy-tamoxifen alters the cholesterol metabolism leading to the accumulation of free sterols, and in turn, MM cell death both in vitro and in vivo [13]. Importantly, they appear to be non-toxic in normal cells at concentrations that would be cytotoxic in cancer cell lines [14,15]. Finally, the 5,6-ECs are unreactive towards neutrophiles, indicating that they are not alkylating agents [16,17]. Taken together, these data demonstrated the potential importance of cholesterol metabolism in the pharmacology and/or therapeutic effects of anti-tumor drugs.

The aim of our study was to investigate the ability of 5,6 α-EC and 5,6 β-EC to trigger human myeloma cells death. Herein, we describe for the first time the anti-tumor activity of the two oxysterols against human myeloma cell lines (HMCLs) and primary MM cells purified from patients via oxiapoptophagy. Our data highlight the pharmacological activity of 5,6 α-EC and 5,6 β-EC alone or in combination with existing treatments for developing efficient anti-MM therapies.

## 2. Materials and Methods

### 2.1. Drugs

The 5,6 α-EC and 5,6 β-EC isomers were kindly provided by Dr. Gérard Lizard (University of Bourgogne Franche-Comté, Dijon, France).

### 2.2. Cell Lines and Culture

JJN3, U266 HMCLs, and the bone marrow normal stromal HS-5 cell line were obtained from Leibniz Institute DSMZ (Brünswick, Germany). They have been described previously [18]. HMCLs were cultured in RPMI 1640 medium supplemented with 10% heat-inactivated fetal bovine serum (FBS, PAN-Biotech, Aidenbach, Germany), 2 mM L-glutamine, and penicillin-streptomycin (PAN-Biotech, Aidenbach, Germany) at 37 °C and 5% CO_2_. HS-5 cells were maintained in complete DMEM medium. Some of HCML characteristics are presented in the Table S1.

### 2.3. Primary Cells Purification from MM Patients

The study was approved by the local ethics committee of our institution (Clinical Research Ethics Committee of La Rabta Hospital, Tunis, Tunisia) and written informed consent was obtained from each patient (Table S2). Bone marrow (BM) samples were freshly aspirated from the sternum of five patients (P1-P5) diagnosed with MM, affiliated to Aziza Othmana Hospital of Tunis. Two to four mL of BM samples were diluted in phosphate-buffered saline (PBS, Sigma-Aldrich, St. Louis, MO, USA), and Ficoll gradient was performed. BM nuclear cells (BMNCs) layer was next carefully collected and washed twice in PBS. For patient P5, BMNCs were analyzed by triple staining with anti-CD38-PE (phycoerythrin, BD Biosciences, San José, CA, USA), -CD138-APC (allophycocyanin, BD Biosciences, Franklin Lakes, NJ, USA) and annexin V-FITC (fluorescein isothiocyanate, Invitrogen, Carlsbad, CA) compounds by flow cytometry with an BD FACSCanto II (BD Biosciences, Franklin Lakes, NJ, USA). For the patients P1-P4, CD138+ malignant plasma cells were isolated from BMNCs and maintained in complete RPMI 1640 medium. The cells were then incubated with CD138-microbeads, passed through LS columns and MiniMACS Separator (Miltenyi Biotec, Bergisch Gladbach, Germany).

### 2.4. MTT Assay

An MTT (3-[4,5-dimethylthiazol-2-yl]-2,5 diphenyl tetrazolium bromide) assay (M5655, Sigma-Aldrich, Saint-Louis, MO, USA) was used to measure the loss of viability of treated cells according to the manufacturer’s instructions. HMCLs or HS-5 cells were plated in 96-well culture plates for 24 h at the density of 2 × 10^5^ cells/mL/well. Then the MTT test was carried out after a 24 to 72 h period of treatments with 5,6 α-EC or 5,6 β-EC with concentrations ranging from 5 to 80 µg/mL. In each experiment, 0.4% ethanol (EtOH) was used as vehicle control. CD138+ primary human myeloma cells were also plated in 96-well culture plates and incubated with 10–80 µg/mL of 5,6 α-EC or 5,6 β-EC for 24 h. The experiment was performed using three replicates for each culture condition and repeated three times. Cell viability was determined by measuring the absorbance with a microplate reader (EXL800, BioTek, Winooski, VT, USA).

The combinations 5,6 α-EC plus 5,6 β-EC (IC_25_ or IC_50_) and 5,6-ECs with bortezomib (BTZ, CAS 179324-69-7, Calbiochem, San Diego, CA, USA) at the indicated doses were also tested. The IC_50_ and IC_25_ (index of cytotoxicity) that are the drug concentrations that kill 50% or 25% of the cells, respectively, after a 48 h-treatment, was calculated with the Prism software (v8.0, GraphPad) and verified with the CompuSyn software (http://www.combosyn.org, accessed on 21 July 2021). This software was also used to calculate the Chou–Talalay combination index (CI).

### 2.5. Assessment of Apoptosis

#### 2.5.1. Cell Cycle Analysis

To determine cell cycle distribution, cells were stained using propidium iodide (PI) and explored by flow cytometry. Following a period of culture of 24 h, JJN3 and U266 cells (2 × 10^5^ cells/mL/well) were incubated with 20–80 µg/mL of 5,6 α/β-ECs for 24–72 h. Cells were next fixed in 70% EtOH at −20 °C for 1 h and incubated with 500 µL of FxCycle PI/RNase staining solution (F10797, Molecular Probes, ThermoFisher Scientific, Waltham, MA, USA) for 30 min. At least, 10^4^ events were gated for each experiment using a BD FACSCanto II (BD Biosciences, Franklin Lakes, NJ, USA). Data were analyzed using the BD FACSDiva 7 software (BD Biosciences, Franklin Lakes, NJ, USA).

#### 2.5.2. Annexin V-Apoptosis Detection Assay

A double staining with annexin V-FITC/PI was used for evaluating the percentage of apoptotic cells induced by each treatment with the Annexin V/Dead cells apoptosis kit (BMS500-FI-300, Invitrogen, Carlsbad, CA, USA). JJN3 and U266 cells were seeded in 24-well plates at the density of 2 × 10^5^ cells/mL, preincubated for 24 h period, then treated with the drugs as described before. CD138+ human myeloma cells were plated in 24-well plates and treated for 24 h with 10–40 µg/mL of 5,6 α-EC or 5,6 β-EC. The cells were then stained with annexin V-FITC/PI according to the manufacturer instructions. At least 10^4^ events were analyzed for each sample using the BD FACSCanto II flow cytometer (BD Biosciences, Franklin Lakes, NJ, USA). Data were processed with the BD FACSDiva 7 software (BD Biosciences, Franklin Lakes, NJ, USA). Four cell sub-populations were evaluated: viable cells (AV–/PI–), early apoptotic cells (AV+/PI–), late apoptotic and/or secondary necrotic cells (AV+/PI+), and necrotic/damaged cells (AV–/PI+).

#### 2.5.3. Analysis of Nuclear Morphology by Hoechst 33,342 Staining

The nuclear morphology of control and 5,6 α-EC or 5,6 β-EC-treated cells was analyzed by fluorescence microscopy after staining with 1 µg/mL of Hoechst 33,342 (B2261, Sigma-Aldrich, Saint Louis, MO) [19]. Almost 4 × 10^4^ cells were applied to glass slides by 5 min cytocentrifugation with a cytospin Shandon 4 (Thermo Fisher Scientific, Waltham, MA). Slides were mounted with the Dako fluorescent mounting medium (S302380, Dako, Copenhagen, Denmark) and observed under an Olympus BX53 fluorescence microscope (Olympus Corporation, Tokyo, Japan).

### 2.6. Effector Caspase 3/7 Activity

To evaluate the effect of 5,6 α/β-ECs on caspase 3/7 activity, the cleavage of the Ac-DEVD substrate was measured using the CellEvent caspase 3/7 green flow cytometry assay kit (C10740, Molecular Probes, Eugene, OR, USA) [20]. JJN3 and U266 cells were seeded at a density of 2 × 10^5^ cells/mL/well in 24-well culture plates for 24 h and treated with 20–80 µg/mL of 5,6 α/β-ECs or with 500 μM H_2_O_2_ as positive control for 24–72 h. Next, cells were processed according to the manufacturer instructions. At least 10^4^ cells per sample were acquired with BD FACSCanto II and data were analyzed with the BD FACSDiva 7 software (BD Biosciences, Franklin Lakes, NJ, USA).

### 2.7. Measurement of Intracellular ROS and Effect of ROS Production on MM Cell Death

The overproduction of intracellular ROS was measured with dihydroethidium (DHE) staining (D11347, ThermoFisher Scientific, Waltham, MA, USA). JJN3 and U266 cells were seeded at a density of 2 × 10^5^ cells/mL/well into 24-well plates and incubated for 24 h before treatments with 20–80 µg/mL of 5,6 α/β-ECs or with 250 or 500 μM H_2_O_2_ as positive controls. Cells were further incubated with 2 µM DHE for 15 min at 37 °C. The production of O^2−^ was monitored by oxidized DHE that exhibits an orange/red fluorescence [11]. The signal was collected from a minimum of 10^4^ events for each sample using the BD FACSCanto II flow cytometer (BD Biosciences) and data were processed with the BD FACSDiva 7 software (BD Biosciences).

JJN3 or U266 cells were plated for 24 h in 24-well plates (2 × 10^5^ cells/well), treated with 5,6 α-EC or 5,6 β-EC (20–80 µg/mL) alone or after a 2 h-pretreatment with 400 µM vitamin E (Vit E, #258024, Sigma-Aldrich, Saint Louis, MO, USA). After 24 or 48 h of treatment, cells were stained with 1 µg/mL PI (#P4170, Sigma-Aldrich, Saint Louis, MO, USA). At least, 10^4^ events per sample were acquired and analyzed by flow cytometry BD FACSCanto II. Data were processed using BD FACSDiva 7 software (BD Biosciences, Franklin Lakes, NJ, USA).

### 2.8. Measurement of Transmembrane Mitochondrial Potential (Δψ_m_)

To evaluate ΔΨ_m_ variations under 5,6 α/β-ECs treatment, the lipophilic fluorescent dye 3,3′-dihexyloxacarbocyanine iodide DiOC_6_-(3) (D273, Invitrogen, Carlsbad, CA, USA) was used [21]. JJN3 and U266 cells were seeded at the density of 2 × 10^5^ cells/mL/well in 24-well plates and incubated for 24 h. Afterward, cells were treated with 20–80 µg/mL of 5,6 α/βECs or with 500 μM H_2_O_2_ serving as a positive control for the experiments. Cells were next incubated with 40 nM DiOC_6_-(3) for 15 min at 37 °C. Mitochondrial depolarization was assessed by a decrease of green fluorescence collected by the BD FACSCanto II flow cytometer (BD Biosciences). A minimum of 10^4^ events per sample were acquired and samples were carried out in triplicate. The BD FACSDiva 7 software was used for data analysis (BD Biosciences, Franklin Lakes, NJ, USA).

### 2.9. Analysis of Autophagy

#### 2.9.1. Detection of Autophagosomes

The activation of autophagy was assessed by the examination of autophagosomes formation with the Autophagy Assay Kit (MAK138, Sigma-Aldrich, Saint Louis, MO, USA). In brief, U266 cells were seeded in 96-well plates with 5,6-ECs (20 or 40 µg/mL) for 20 h in 37 °C or the vehicle. The autophagosome fluorescent reagent was then added to each well and incubated at 37 °C for 15 min. U266 cells were next observed under an Olympus BX53 fluorescent microscope (Olympus corporation, Tokyo, Japan) (×40, magnification).

#### 2.9.2. Determination of p62 Expression by Indirect Immunofluorescence

U266 cells were treated with vehicle, 5,6 α-EC (40 µg/mL) or 5,6 β-EC alone (20 or 40 µg/mL). The cells were cytospun on superfrost glass slides, fixed in 4% paraformaldehyde, and permeabilized in 0.5% Triton-X100. The slides were then stained with an anti-p62 (SQSTM1) as primary antibody (Ab), and with Alexa Fluor 633- (in red) conjugated goat anti-mouse IgG as secondary Ab (Invitrogen, Carlsbad, CA, USA). Slides were counterstained with DAPI in blue (Molecular Probes, Eugene, OR, USA) and observed with a confocal microscope (Fluoview FV100, Olympus, Tokyo, Japan).

#### 2.9.3. Effects of Autophagy Inhibition or Triggering on MM Cell Death and Apoptosis

U266 cells were seeded in 24-well plates (2 × 10^5^ cells/well) for 24 h, treated with 5,6 α-EC or 5,6 β-EC (20–80 µg/mL) alone or in combination with 5 µM rapamycin (#R0395) or 10 µM of 3-methyladenine (3-MA) (#M9281), as autophagy inducer and inhibitor, respectively, all from Sigma-Aldrich (Saint Louis, MO, USA). After 24 h of treatment, cells were stained with 1 µg/mL PI for cell death assessment. At least, 10^4^ events per sample were acquired and analyzed by flow cytometry BD FACSCanto II. Data were processed using BD FACSDiva 7 software (BD Biosciences, Franklin Lakes, NJ, USA).

U266 cells were seeded in 24-well plates (2 × 10^5^ cells/well) for 24 h, pre-treated (or not for control) with 50 nM bafilomycin A1 (BafA1, 19-148, Merck, Darmstadt, Germany) then treated with 5,6 β-EC (20 or 40 µg/mL). After 24 h of treatment, the cells were stained with annexin V-FITC/PI for apoptosis assessment as described before. At least, 10^4^ events per sample were acquired and analyzed by flow cytometry with the CytoFlex cytometer and the CytExpert software (Beckman Coulter, Pasadena, CA, USA).

### 2.10. Western Blotting

Cell lysis was performed by incubating cells on ice for 30 min in a lysis buffer containing 1% NP-40, 100 mM Tris-HCl and a cocktail of protease inhibitors. Cell lysates were cleared by centrifugation at 20,000× *g* for 15 min. Proteins (50 µg) were separated by SDS-PAGE, and blotted onto nitrocellulose membranes (Bio-Rad, Hercules, CA, USA). After blocking non-specific binding sites, membranes were incubated with primary antibodies according to the manufacturer instructions. The following antibodies were used: anti-LC3B (#51520) from abcam (Cambrigde, UK); anti-caspase-3 (#9662), -PARP (#9532) and -β-actin (#4970) from Cell Signaling Technology (Danvers, MA, USA). Membranes were incubated with horseradish peroxidase (HRP)-conjugated goat anti-mouse (sc-2005, Santa-Cruz Biotechnology, CA, USA) or HRP-conjugated goat anti-rabbit (#6721, abcam, Cambrigde, UK). Membranes were revealed with a chemiluminescence detection reagent (Western Bright Quantum HRP substrate, Advansta, San Jose, CA, USA) using a ChemiDoc XRS+ imaging system (Bio-Rad).

### 2.11. Statistical Analyses

In vitro experiments were performed three times with samples in triplicate (*n* = 3). Representative results are shown as means ± standard deviation (SD). Statistical analyses were carried out with the two-tailed unpaired Student’s t-test using the Excel software. The differences between two experimental groups were considered to be statistically significant when the obtained *p*-value was <0.05. To avoid unnecessarily loading the figures, the statistical data are gathered in additional tables in the Supplementary Information file.

## 3. Results

### 3.1. 5,6 α-EC and 5,6 β-EC Exhibit Cytotoxic Activities on JJN3 and U266 Cell Lines

The cytotoxic activities of 5,6 α-EC and 5,6 β-EC were assessed by multiple in vitro standard tests: MTT assay, FDA (fluorescein diacetate) assay, Trypan blue exclusion, PI staining, and cytometry sorting on a panel of MM cell lines belonging to various molecular subtypes (Table S1). MTT assays were performed after 24, 48, or 72 h treatments with 5,6 α-EC or 5,6 β-EC isomers at various concentrations (5–80 μg/mL). Most of cells were responsive to both isomers as observed on the viability curves (Figure S1). We next focused on U266 and JJN3 cells that were representative.

**Figure 1:**
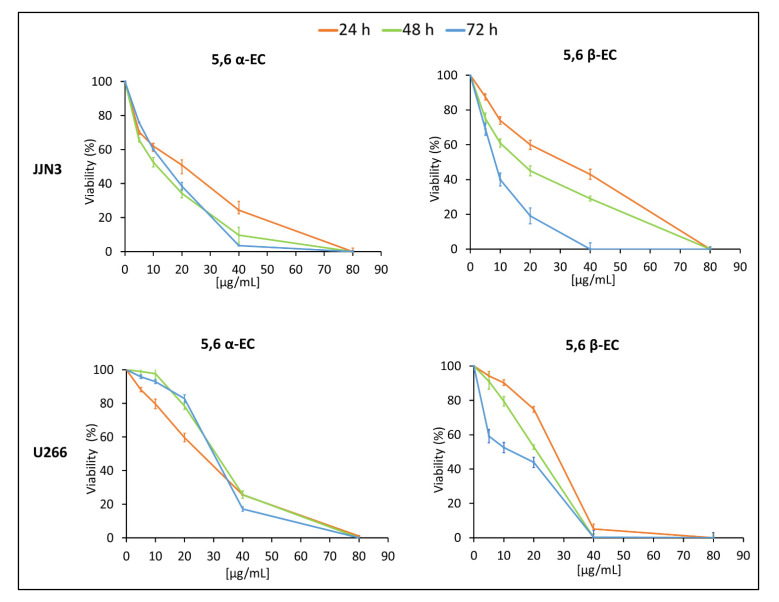
Evaluation of 5,6-ECs-induced cytotoxic effects on HMCLs. JJN3 and U266 cells were seeded in 96-well plates at the density of 2 × 10^5^ cells/well for 24 h. Then, cells were cultured with 5–80 μg/mL 5,6 α-EC or 5,6 β-EC for 24–72 h and their viability assayed using a colorimetric MTT assay. Reported values are means ± SD from three independent experiments. No statistically significant difference between control (no treatment) and vehicle (EtOH-treatment) was noticed.

The results revealed that 5,6 α-EC and 5,6 β-EC compounds significantly decreased HMCLs viability in a dose-dependent manner (Figure 1). JJN3 cells are more sensitive than U266 cells to both α and β isomers according to the calculated IC_50_ values at 48 h (11 and 14 µg/mL, respectively, for JJN3 vs. 31 and 21 µg/mL, respectively, for U266 cells (Table 1). The 5,6 α-EC cytotoxic effect was maximal 24 h-post treatment for both cell lines whereas 5,6 β-EC showed a time-dependent cytotoxic effect (Figure 1). Accordingly, the calculated IC_50_ decreased in a time dependent manner for JJN3 cells from 21 µg/mL (48 h) to 12 µg/mL (72 h) and for U266 cells, from 14 µg/mL (48 h) to 7 µg/mL (72 h) (Table 1). The cytotoxic effects of 5,6-ECs on JJN3 and U266 were confirmed with the FDA viability assay (Figure S2). An anti-proliferative effect of both compounds on JJN3 and U266 cells was also visualized under an inverted-phase contrast microscope (Figure S3).

### 3.2. 5,6 α-EC and 5,6 β-EC Isomers Induce Apoptosis

**Figure 2:**
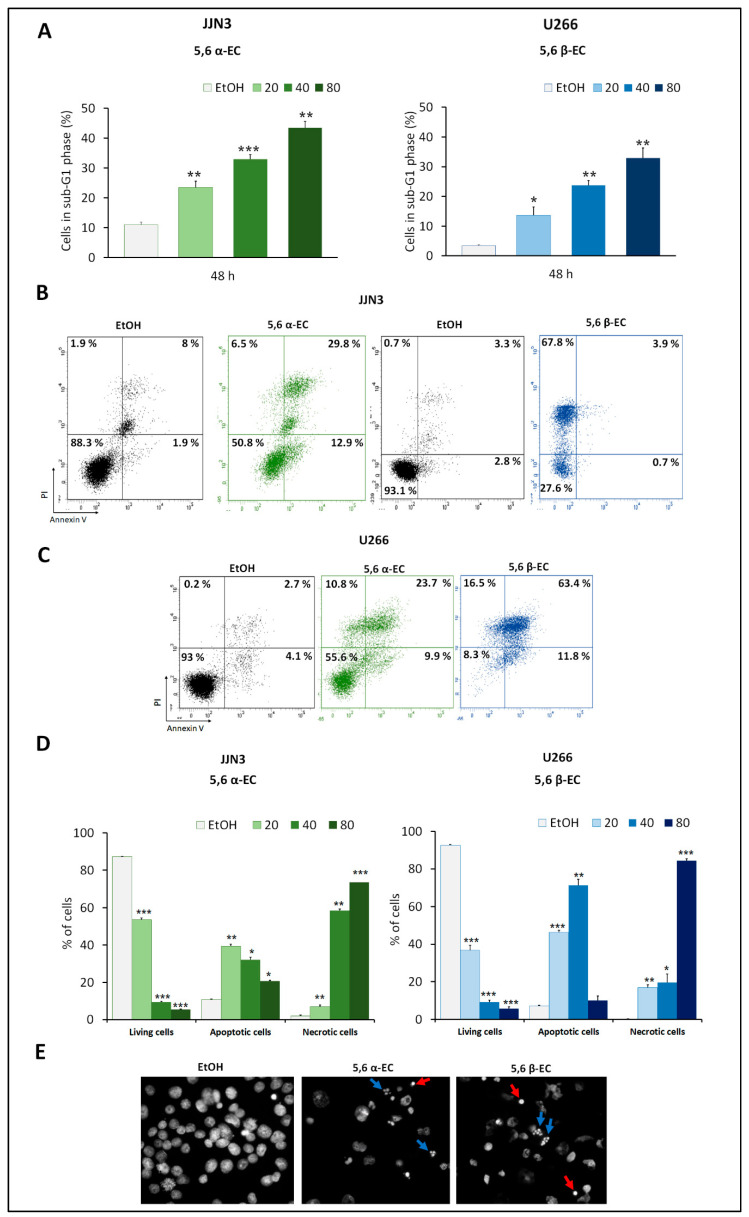
5,6-ECs induce apoptosis of HMCLs. (**A**) JJN3 and U266 cells were seeded at the density of 2 × 10^5^ cells/well in 24-well plates for 24 h and treated for 48 h with 20–80 μg/mL 5,6 α-EC or 5,6 β-EC. To quantify cells in the sub-G1 cell cycle phase after treatment, cells were fixed in EtOH 70% and stained with PI. DNA content was measured by flow cytometry. The histograms represent the percentages of JJN3 or U266 cells in the sub-G1 phase as means ± SD (Table S3). (**B**,**C**) To investigate 5,6ECs-induced apoptosis, cells were harvested and stained with annexin V/PI. Data were acquired by flow cytometry. Living cells (annexin V–/PI–), apoptotic cells (annexin V+/PI– and annexin V+/PI+) and necrotic cells (annexin V–/PI+) were recorded. Cytometry profiles are presented for JJN3 cells treated with 20 µg/mL 5,6-ECs (**B**) and U266 cells treated with 40 µg/mL 5,6-ECs. (**D**) The percentages of each population as means ± SD are reported in the histograms for JJN3 and U266 cells. * *p* < 0.05; ** *p* < 0.01; *** *p* < 0.001 with the *t*-test (Tables S4 and S5). No statistically significant difference between control and vehicle was noticed. (**E**) U266 cells were treated for 24 h with 40 μg/mL 5,6 α-EC or 5,6 β-EC. After fixation in 2% PFA, cells were cytospun on slide glasses. Nuclei were stained by Hoechst 33,342 and visualized by fluorescence microscopy. Red arrows indicate cells with condensed chromatin; blue arrows cells with fragmented nuclei (×400, magnification).

To investigate whether 5,6 α-EC or 5,6 β-EC impacted cell cycle progression, HMCLs were treated with various concentrations of each 5,6-EC isomers (20–80 µg/mL) for 24 or 48 h. Cell cycle distribution was assessed after PI staining and flow cytometry sorting. For each treatment, cytometry profiles of both JJN3 and U266 cells indicated the emergence of a population of cells having a sub-G1 DNA content representative of apoptotic cells (Figure 2A). Indeed, after a 48 h-treatment, this percentage significantly increased in JJN3 cells of (+ 21.87% and + 3.5%) and in U266 (+ 6.86% and + 20.33%) for 40 µg/mL 5,6 α-EC or 5,6 β-EC, respectively (Figure 2A, Table S3).

AnnexinV/PI staining and cytometry analyses also confirmed that an apoptotic mode of cell death was triggered by 5,6 α-EC and 5,6 β-EC in JJN3 and U266 HMCLs. As exemplified for JJN3 cells, after a treatment with 20 µg/mL 5,6 α-EC for 48 h, 42.7% of cells are apoptotic (12.9% annexin V+/PI– and 29.8% of annexin V+/PI+) (Figure 2B). In the same experimental conditions, after treatment with 40 µg/mL 5,6 β-EC, 75.2% of U266 cells are apoptotic (63.4% annexin V+/PI–, 11.8% annexin V+/PI+), (Figure 2C). Notably, apoptotic cells were also detected in U266 cells treated with 5,6 α-EC and 5,6 β-EC for 24–72 h (Figure 2D, Tables S4 and S5).

Cytometry data showed that 5,6-ECs-treated cells went through apoptosis, and necrosis when treatments duration was extended, and drug doses increased. Thus, both 5,6-ECs compounds triggered an apoptotic cell death; the β isomer being more potent than the α isomer and JJN3 cells more sensitive than U266 cells confirming the data obtained with the viability assays.

Finally, Hoechst 33,342 staining was used to visualize the morphological changes induced by 5,6-ECs treatments in MM cells. Images revealed remarkable morphological changes giving rise to condensed or fragmented nuclei (apoptotic bodies) and diffused nuclei (in necrotic cells) (Figure 2E).

### 3.3. 5,6 α-EC and 5,6 β-EC Isomers Activate the Mitochondrial Intrinsic Apoptotic Pathway

**Figure 3:**
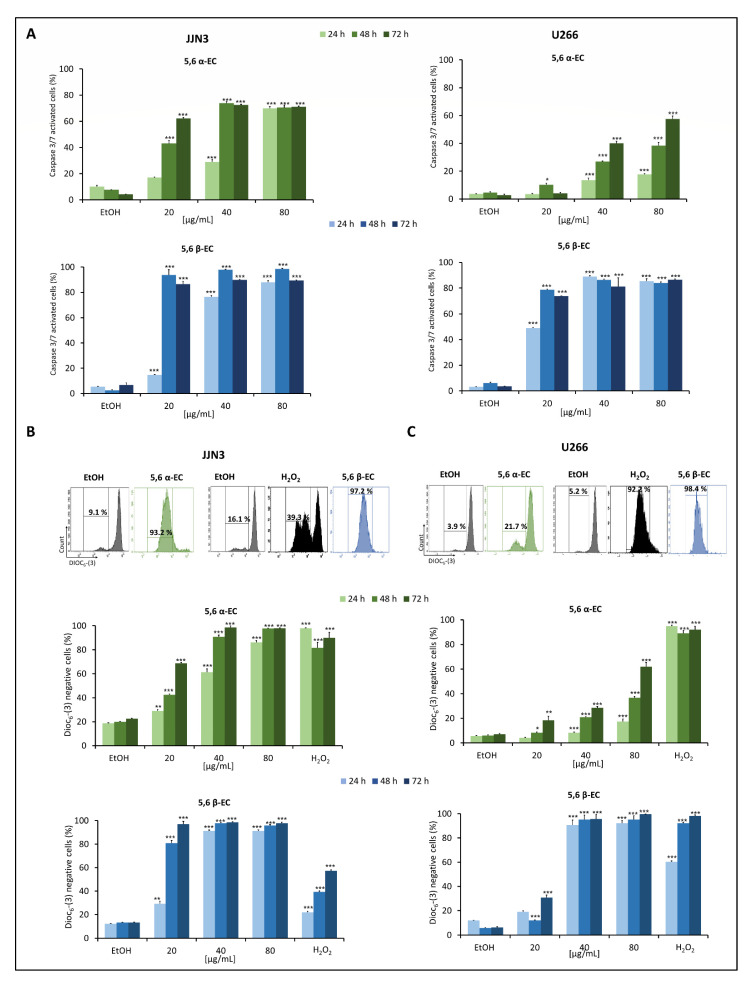
The apoptotic intrinsic pathway is triggered by 5,6-ECs treatment in HMCLs. (**A**) JJN3 and U266 cells were seeded in 24-well plates at a density of 2 × 10^5^ cells/well for 24 h and treated with 5,6 α-EC or 5,6 β-EC (20–80 μg/mL) for 24–72 h. Caspase 3/7 enzymatic activity was detected by flow cytometry in both cell lines. The percentage of cells with activated caspase 3/7 was presented in histograms as the mean ± SD (Table S6). The percentages of DiOC_6_-(3) negative (with depolarized mitochondria) JJN3 (**B**) or U266 (**C**) cells obtained by flow cytometry are showed in histograms as the means ± SD. H_2_O_2_ (500 µM) was used as a positive control. No statistically significant difference between control and vehicle (EtOH) was noticed. * *p* < 0.05; ** *p* < 0.01; *** *p* < 0.001 with the *t*-test (Table S7).

To further characterize the type of apoptosis triggered by 5,6 α/β-EC, we investigated the executioner caspase 3/7 activities in the same settings. The enzymatic activities of caspase 3/7significantly increased in a dose and time-dependent manner in 5,6 α-EC or 5,6 β-treated JJN3 and U266 cells (Figure 3A, Table S6).

In agreement with these observations, the treatments with 5,6 α-EC (40 µg/mL) or 5,6 β-EC (20 or 40 µg/mL) led to the cleavage and activation of caspase 3 and/or the cleavage of PARP (Figure S3). Mitochondrial depolarization (ΔΨm) is considered as the point-of-no-return of apoptosis and is essential for the activation of caspase 3/7 [22]. Hence, ΔΨm was measured by flow cytometry after DiOC_6_-(3) staining of JJN3 and U266 cells after exposure to 20–80 µg/mL 5,6 α-EC or 5,6 β-EC for 24–72 h. DiOC_6_-(3) being selective for the mitochondria of living cells, a decrease in ΔΨm is representative of mitochondria collapse. We used as a positive control a hydrogen peroxide (H_2_O_2_)-treatment (500 µM for 24 h). As shown in Figure 3B,C, H_2_O_2_-treatment significantly decreased the fluorescence intensity correlating with an increase of the fraction of cells having a ΔΨm loss. For example, JJN3 cells showed a significant loss of ΔΨm after a 20 µg/mL 5,6 α-EC-treatment (+ 84.1%) or 5,6 β-EC (+ 81.1%) for 48 h. In fine, 5,6 α-EC and 5,6 β-EC compounds induced a significant dose-dependent depolarization of mitochondria compared to vehicle-treated cells confirming that both isomers triggered the mitochondrial-dependent apoptotic pathway in MM cells (Table S7). Moreover, 5,6 β-EC was confirmed more efficient than 5,6 α-EC on both cell lines (Figure 3).

### 3.4. 5,6 α-EC and 5,6 β-EC Increased ROS Production in JJN3 and U266 Cells

**Figure 4:**
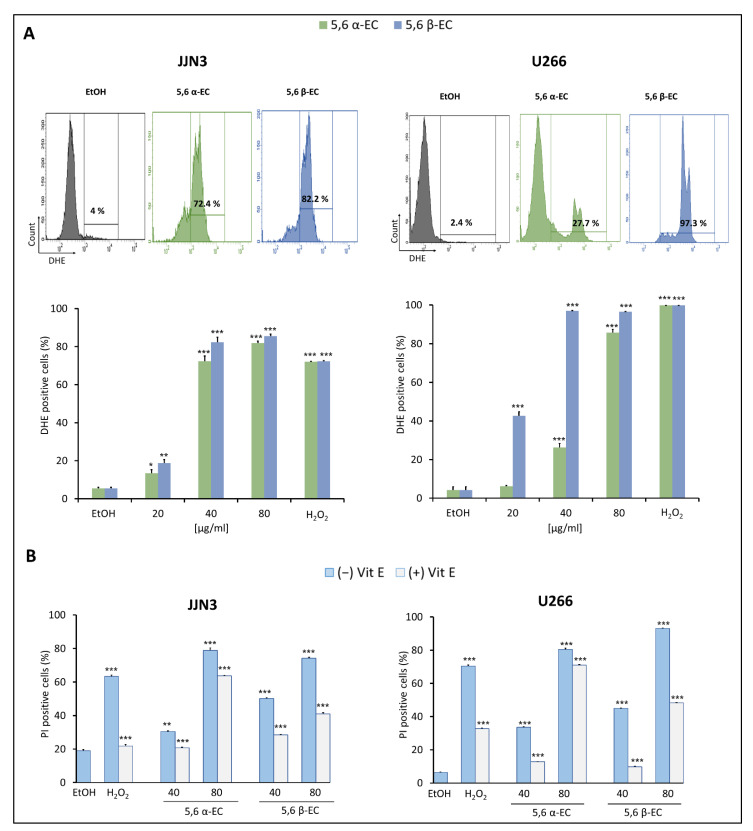
ROS overproduction induced cell death of 5,6 ECs-treated HMCLs. (**A**) JJN3 and U266 cells were cultured in 24-well plates (2 × 10^5^ cells/well) for 24 h. Then, cells were treated with 20–80 μg/mL of 5,6 α-EC or 5,6 β-EC for 24 h or with 500 µM H_2_O_2_ used as a positive control. ROS overproduction was evaluated by flow cytometry (upper part). The percentages of DHE+ cells (overproducing superoxide anion O^2−^) are indicated by the histograms (lower part). (**B**) Cells were treated or not with 400 µM Vit E for 2 h then, treated for 24 h with 5,6 α-EC or 5,6 β-EC (40–80 µg/mL). The inhibition of death induced by 5,6-ECs was evaluated by the percentage of PI+ HMCLs presented as means ±SD. Statistical *t*-tests were used to calculate the *p*-values between 5,6 α/β-ECs-treated cells vs. vehicle-treated cells or between 5,6 α/β-ECs- vs. 5,6 α/β-ECs + Vit E-treated cells (* *p* < 0.05; ** *p* < 0.01; *** *p* < 0.001) (Table S8). No statistically significant difference between control and vehicle (EtOH) was noticed.

To determine the ability of 5,6 α-EC and 5,6 β-EC to induce an oxidative stress, we evaluated the production of ROS by flow cytometry after DHE staining of treated cells. Indeed, the percentage of DHE+ (i.e., producing superoxide anions) JJN3 cells significantly increased from 4% (vehicle) to 72.4% and 82.2% when cultured with 40 µg/mL 5,6 α-EC or 5,6 β-EC, respectively (Figure 4A). In agreement, ROS levels also increased in U266 cells cultured in the same conditions from 2.4% (vehicle) to 27.7% and 97.3%, respectively. After a 24 h-treatment with 5,6 α-EC or 5,6 β-EC (20–80 µg/mL), the percentage of DHE+ JJN3 and U266 cells significantly increased in a dose-dependent manner.

Moreover, we used α-tocopherol (or Vit E) to prevent 5,6 ECs-induced ROS. Cell death was evaluated in HMCLs by PI staining after a 24 h-treatment with 40 or 80 µg/mL 5,6 α-EC or 5,6 β-EC in the presence or the absence of Vit E. Results showed that Vit E remarkably reduced 5,6ECs-induced cell death (Figure 4B, Figure S5, Table S8). For example, the percentage of PI+ U266 cells significantly decreased from 33.5% with 40 µg/mL 5,6 α-EC and 40.1% with 40 µg/mL 5,6 β-EC, to 12.8% and 9.8% in combination with Vit E (400 µM), respectively. Flow cytometry data showed similar results for JJN3 cells supporting that Vit E prevented the production of ROS and the induction of an oxidative stress leading to cell death.

Finally, we inhibited ROS generation with a NAC pre-treatment (1 mM for 12 h) and treated U266 cells with 20 or 40 µg/mL 5,6 β-EC for 24 h. Apoptotic cells (annexin V+) were analyzed by flow cytometry. As presented Figure S6, the inhibition of ROS generation protected cells from 5,6-ECs-induced apoptosis. These findings confirmed that oxidative stress is an important event for 5,6-ECs-induced MM cell death and in particular, apoptosis.

### 3.5. 5,6 α-EC and 5,6 β-EC Induce Autophagy in Myeloma Cells

**Figure 5:**
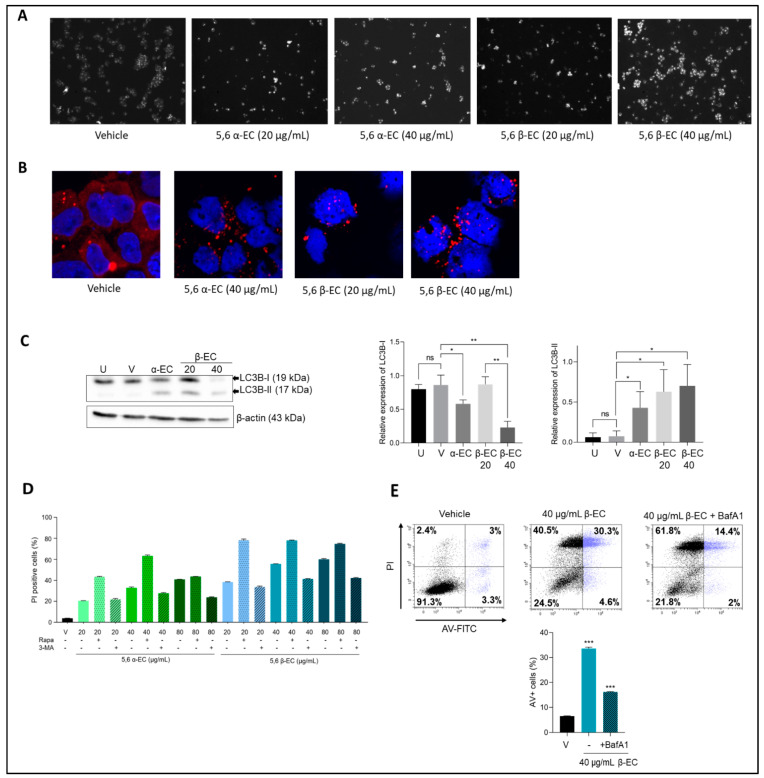
5,6-ECs induce an autophagy-mediated cell death. (**A**) U266 cells were treated with vehicle or 5,6 α/β-EC (20, 40 µg/mL) for 20 h. Cells were then stained with an autophagosome specific fluorescent probe and analyzed with an Olympus BX53 fluorescent microscope (× 40, magnification) (**B**) p62 expression was analyzed by IF in EtOH- and 5,6-ECs-treated cells. We used a primary Ab against p62, and a goat Alexa Fluor 488-conjugated anti-rabbit IgG as secondary Ab. Slides were counterstained with DAPI and analyzed with a confocal microscope (Fluoview FV100, Olympus, CA, USA) (× 180, magnification). (**C**) U266 cells were untreated (U), vehicle-treated (E) or with 5,6 α-EC (40 µg/mL) or 5,6 β-EC (20 or 40 µg/mL). Within 24 h, whole-cell proteins were extracted, separated by SDS-PAGE, and transferred onto membranes then incubated with anti-LC3B or anti-β-actin (as a control) antibodies. Protein levels were estimated by densitometry and collected data from three independent experiments were presented in histograms (means ± SD). (**D**). The modification of autophagic flux was evaluated by flow cytometry. U266 cells were seeded into 24-well plates for 24 h. The cells were treated with 5,6 α-EC or 5,6 β-EC alone or in combination with 5 µM rapamycin or 10 µM 3-MA for 24 h. The cells were stained with PI and PI positive cells were recorded. At least 10^4^ events were gated. The experiment has been done once with triplicate samples. (**E**) U266 cells were pre-treated with BafA1 (50 nM) for 4 h and then treated with 5,6 β-EC (40 μg/mL). The cells were stained with annexin V/IP as described before and sorted. Cytometry profiles were presented together with the percentage of cells within each quadrant. The percentage of annexin V+ cells in each culture condition is presented in the histogram. * *p* <0.05; ** *p* < 0.01; *** *p* < 0.001; ns, not significant with the *t*-test.

To investigate whether 5,6-EC isomers induced autophagy concomitantly to apoptosis, we first assessed the presence of autophagosomes in 5,6-ECs-treated U266 cells stained with a fluorescent autophagosome marker. Microscopic examination of stained cells revealed the presence of autophagosomes. Moreover, their number increased in a dose-dependent manner (Figure 5A). In agreement with a previous report, U266 such as MM cells exhibited a remarkable basal autophagic activity (ref, Milan et al. J Clin Immunol 2016, 36, 18–24). The presence of autophagosomes reminiscent of an active autophagy was also revealed by the confocal microscopic examination of p62 (SQSTM1), the autophagy cargo receptor. As shown Figure 5B, the p62 staining pattern changed from diffuse (control cells) to punctate and cytoplasmic (treated cells) in a dose-dependent manner.

We next studied by western blotting the conversion of microtubule-associated protein light chain 3 (LC3)-I to LC3-II in U266 treated cells to assess the autophagic flux (Figure 5C, Figure S7). Interestingly, a significant decrease of LC3B-I relative level accompanied by a significant increase of LC3B-II relative levels were observed under treatment with 40 µg/mL 5,6 α-EC or 20 or 40 µg/mL 5,6 β-EC. Additionally, the decrease of relative LC3B-I level was significantly dose-dependent when cells were exposed to 20 or 40 µg/mL 5,6 β-EC.

Moreover, rapamycin (an PI3K inhibitor) and 3-methyladenine (3-MA) (an mTOR inhibitor), the most widely used autophagy inducer or inhibitor, were employed to investigate their ability to modify the autophagic flux in 5,6-ECs treated MM cells. Notably, flow cytometry analysis of PI-stained cells showed that the 5,6-ECs-induced cell death was enhanced in the presence of rapamycin and reduced in the presence of 3-MA (Figure 5D). Moreover, BafA1, an inhibitor of autolysosomes formation, also protected U266 cells from 5,6 β-EC-induced apoptosis (Figure 5E). These results confirmed that all used chemicals modified the autophagic flux in treated MM cells, which is supporting that both isomers triggered an autophagic cell death in MM cells.

### 3.6. 5,6 α-EC and 5,6 β-EC Reduce Cell Viability and Induce Apoptosis in Primary MM Cells

**Figure 6:**
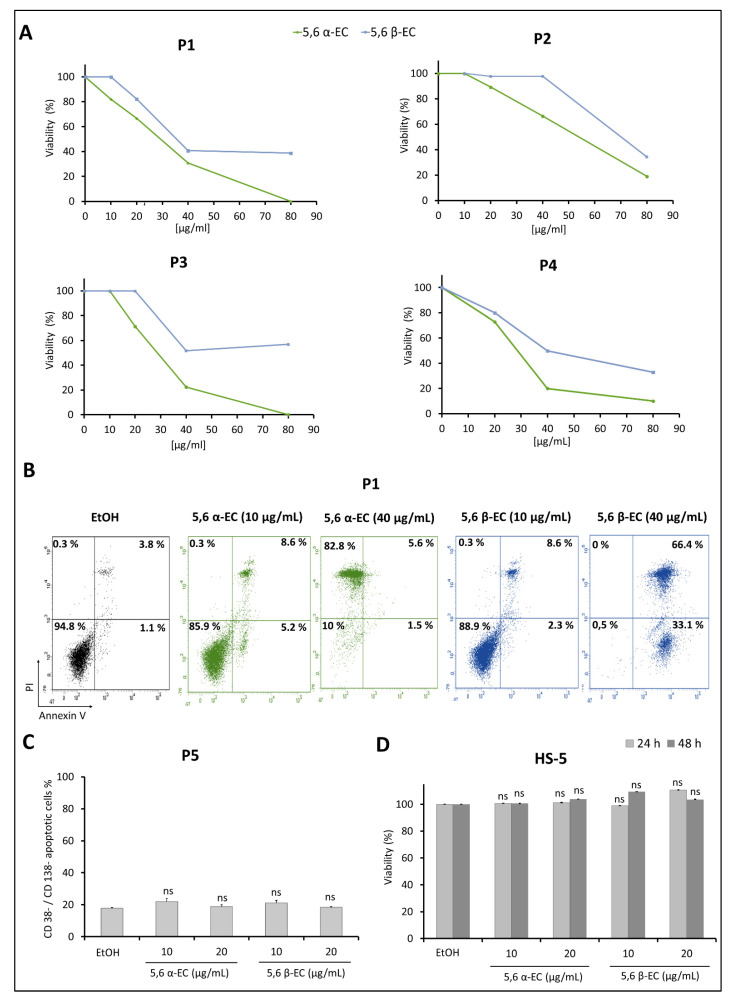
5,6-ECs display selective cytotoxic and apoptotic activities on human primary malignant plasma cells. (**A**) Cytotoxic activity of compounds was assessed on patient malignant cells of P1, P2 and P3 by MTT and for P4 cells by Trypan blue. For this, mononuclear cells (BMNCs) were purified from bone marrow and cell sorting using CD138 microbeads was assessed for P1-P4. CD138+ malignant plasma cells were cultured for 40 h in the absence or presence of 10–80 μg/mL of 5,6 α-EC or 5,6 β-EC. (**B**) Annexin V staining and flow sorting was used to detect the apoptotic population in sorted CD138+ cells of P1 after treatments with 10 or 40 µg/mL 5,6 α-EC or 5,6 β-EC. Flow cytometry profiles for all cell compartments (annexin V–/PI–, annexin V+/PI–, annexin V+/PI+ and annexin V-/PI+) with the corresponding percentages are presented. (**C**) BMNCs of P5 were treated with 10 µg/mL or 20 µg/mL of 5,6 α-EC or 5,6 β-EC for 24 h then labeled simultaneously for CD38, CD138 and annexin V. Annexin V+/CD38–/CD138– population was next evaluated by flow cytometry and presented in the histograms as means ±SD. (**D**) The effect of 5,6-ECs on HS-5 human non-tumor mesenchymal stromal cells was also evaluated using an MTT assay after 24 or 48 h of treatment. Data are presented as the means ±SD. No statistically significant difference between control and vehicle (EtOH) was noticed; ns, not significant.

Sorted CD138+ malignant plasma cells isolated from BMNCs of four MM patients (P1 to P4) were exposed to various concentrations (10–80 μg/mL) of 5,6 α-EC or 5,6 β-EC and MTT assays were performed. Due to the limited number of purified MM cells, only the counting with Trypan blue dye was performed with CD138+ cells from P4 patient. MTT results revealed that both molecules strongly reduced cell viability of CD138+ cells from all patients tested (Figure 6A). Indeed, under exposure with the highest dose of 5,6 α-EC, the viability of CD138+ cell was reduced by 81% for patient P2 and totally for patients P1 and P3, compared to vehicle control. The response of MM cells from patients was dose dependent. Regarding 5,6 β-EC, the decrease of CD138+ viability reached 59.2% and 48.2% for MM cells of patients P1 and P3, respectively, for a 40 μg/mL-treatment and remained almost constant for the highest dose of 80 μg/mL. An inhibition of 65.7% of the viability was obtained with the dose of 80 μg/mL for the patient P2.

Additionally, Trypan blue counting of MM cells from patient P4 showed a clear dose-dependent decrease of living cells (Figure 6A). Only 10% and 33% of cells remained viable following 80 μg/mL 5,6 α-EC-or 5,6 β-EC-treatment, respectively. For patient P4, we investigated whether the 5,6-ECs cytotoxic effect was exerted through apoptotic cell death. CD138+ cells isolated from patient P4 were treated with 10 or 40 μg/mL 5,6 α-EC or 5,6 β-EC for 40 h and next stained with annexin V/PI. As shown in Figure 6B, both isomers triggered primary cells apoptosis. The percentage of CD138+ cells in early and late apoptosis (annexin V+/PI– and annexin V+/PI+) increased from 4.9% in the control sample to 13.8% and 10.9% in the samples treated with 10 μg/mL of 5,6 α-EC or 5,6 β-EC, respectively. The percentage of apoptotic cells reached 99.5% when they were treated with 40 μg/mL 5,6 β-EC; the cells were almost all necrotic when treated with the same concentration of 5,6 α-EC.

Further, we examined whether apoptosis was induced by 5,6-ECs in non-tumor CD38–/CD138– BMNCs sub-population from patient P5. For that purpose, BMNCs were cultured in the presence of 5,6 α-EC or 5,6 β-EC (10 or 20 μg/mL) for 24 h. Next, triple staining of BMNCs (CD38, CD138, and annexin V) followed by flow cytometry analysis showed that the percentages of annexin V+ non tumor cells were unchanged after 5,6 α-EC, 5,6 β-EC, or vehicle-treatment (18.8% and 18.4% vs. 17.9%) (Figure 6C). Our results indicated a lack of cytotoxicity of the 5,6-ECscompounds against CD38–/CD138– bone marrow cells. Additionally, 5,6 α-EC or 5,6 β-EC were not cytotoxic toward the normal human bone marrow stromal (HS-5) cell line treated with 10 or 20 µg/mL of each isomer for 24 or 48 h as observed by an MTT assay (Figure 6D). We concluded that both molecules exhibited apoptotic features only toward malignant CD138+ plasma cells and were not toxic against other BMNCs and HS-5 mesenchymal stromal cells.

Overall and by contrast with the results obtained with MM cell lines, although each MM patient possesses her/his own sensitivity towards 5,6 α/β-EC compounds, the α isomer seemed more potent for inhibiting tumor cell proliferation and inducing MM cell death.

### 3.7. 5,6 α-EC and 5,6 β-EC Exhibit Synergistic Cytotoxic Effects in MM Cells

Multi-agent therapies are a hallmark of treatment for multiple myeloma. Therefore, we analyzed the combination of the two 5,6-ECs isomers on U266 MM line. Cells were treated with both 5,6 α-EC or 5,6 β-EC with the IC_25_ or IC_50_ corresponding concentrations for 24 and 48 h (Table 1, Figure S8). Cell viability was assessed by MTT assay, and the results are reported in Figure S8. Interestingly, following the combination treatment with the IC_25_ corresponding doses, the cell viability decrease was higher than the sum of the cytotoxic effects taken separately, in a dose-dependent manner. U266 cell death increased from approximately 30% (for each isomer) to 81% (for the combination) after 24 and 48 h of exposure (Figure S8). The trend was also maintained for higher doses (IC_50_). Moreover, the Chou–Talalay combination index (CI) calculated with the CompuSyn software, indicated a strong (notable) synergistic effect. In fact, the CI value was less than 1 with both IC_25_ and IC_50_ treatment doses, supporting favorably a synergistic interaction between 5,6 α-EC and 5,6 β-EC on myeloma cells (Table 2).

### 3.8. 5,6 α-EC and 5,6 β-EC Exhibit Synergistic Cytotoxic Effects with BTZ in MM Cells

Finally, we analyzed a potential synergic effect of both 5,6-ECs with the gold standard drug in MM: bortezomib. We compared anti-tumor effect of the single treatments with 5,6-ECs and the combined treatments 5,6 α-EC/BTZ or 5,6 β-EC/BTZ with an MTT assay. The concentrations of the drugs that were used are indicated in the Table 3, as well as the Chou–Talalay CI. The dual BTZ/5,6-ECs treatment revealed a strong synergistic effect reinforcing the interest of oxysterol compounds for anti-myeloma therapy.

## 4. Discussion

Despite the development of novel successful therapies, MM remains an incurable type of cancer, since relapse and treatment resistance mainly occur with a median overall survival of four years [2]. Investigating lipid metabolism, often deregulated in MM patients, is one way to help developing new treatments and/or improving pre-existing ones [6]. In human body, oxysterols act as important regulators of physiological, physio-pathological and pharmacological processes [23].

Herein, we report for the first time the anti-tumor activities of the 5,6-ECs α and β isomers on HMCLs and primary cells isolated from MM patients. Indeed, a significant decrease of cell proliferation was observed for treated HMCLs and primary cells in a dose-dependent manner. We note that 5,6 β-EC was more efficient on HMCLs, while the 5,6 α-EC seemed more efficient on patients’ malignant cells. Moreover 5,6-ECs α and β isomers demonstrate a high cytotoxicity against JJN3 and U266 cells having various sensitivities towards bortezomib, the gold standard drug [18], whereas they have no effects on normal bone marrow mesenchymal HS-5 cells. Our findings are in agreement with previous data reporting that cholesterol derivatives, including 5,6 α-ECs, suppress proliferation and survival of human breast cancer and mouse skin melanoma cell lines in vitro [24].

The type of cell death induced by 5,6-ECs isomers in myeloma cells was further investigated. Using various assays, we demonstrated that 5,6 α/β-ECs trigger apoptosis in HMCLs and malignant primary cells. The activation of the apoptotic pathway leads to morphological changes such as chromatin condensation and generation of apoptotic bodies (Figure 2E). Cellular alterations culminate with DNA fragmentation and PARP cleavage (Figure S3) conductive to cell death. 5,6 α/β-ECs-mediated apoptosis depend on the activation and the cleavage of caspase 3/7 (Figure S3). In agreement with our findings, Fernandes and coworkers reported that 7-ketocholesterol, an oxidized cholesterol derivative, induces an apoptosis-mediated cell death in chronic myeloid leukemia cell lines [25].

Ex vivo preclinical studies on primary patient-derived tumor cells yield results with a high predictive value towards the clinical outcome. To this end, we sought to examine the effects of 5,6-ECs α and β isomers on CD138+ primary cells purified from four MM patients at diagnosis (Figure 6). The inhibition of proliferation and the induction of apoptosis in response to 5,6-ECs were investigated. Both isomers display a significant cytotoxic activity against CD138+ primary cells isolated from MM patients. This effect was mediated via apoptosis cell death as revealed for patient P4. Moreover, both isomers were not cytotoxic against mesenchymal HS-5 cells (Figure 6D) and patient P5 bone marrow mononuclear cells (CD138–/CD38–) (Figure 6C).

Oxysterols induce apoptosis through both the death receptor or extrinsic pathway and the mitochondrial or intrinsic pathway [26]. We reported that 5,6-ECs treatment cause a high ΔΨm loss in both JJN3 and U266 myeloma lines (Figure 3B,C), indicating that the mitochondrial intrinsic pathway is activated. Alterations of ΔΨm not only result in mitochondrial membrane permeabilization but also in the production of ROS [27]. The 5,6-ECs treatment significantly induces the production of high levels of ROS (especially O^2−^) implicated in HMCLs cell death as revealed by vitamin E cytoprotection (Figure 4B, Figure S4). Our previous data supported the interest to promote an oxidative stress in an anti-MM strategy [18]. In particular, increasing ROS to high levels may provide a unique tool to kill myeloma cells [28]. Indeed, we recently reported that modulating the redox balance of MM cells could be an effective therapy for refractory or post-treatment patients [18].

Besides apoptosis, an autophagic cell death was reported in various cell types treated with oxysterols [29]. The generation of ROS induces a protective mechanism of autophagy in MM cells and if the unfolded protein response is chronically compromised, autophagy results in cell death [30]. We therefore investigated this type of cell death in 5,6-ECs-treated HMCLs and found an increase of autophagosomes correlated with the treatment (Figure 5A). The microtubule-associated protein 1A/B-light chain 3B (or LC3B) is translocated to autophagosomes and the conversion of LC3B-I into LC3B-II is a robust marker of autophagy [31]. Both isomers affect the expression of light chain 3 in increasing the level of the LC3B-II and those of p62, the autophagosome cargo, consistent with an active autophagy (Figure 5B,C). Moreover, both EC isomers modulate the autophagic signaling pathway when cells were pre-incubated with rapamycin or 3-MA. Notably, molecules capable of modulating autophagy are increasingly proposed for therapeutic purposes, including MM [32,33]. For example, betulinic acid was found to induce either apoptotic or autophagic cell death in MM cells [34]. Similarly, Fu and coworkers reported that the activation of the endoplasmic reticulum stress, using the drug tunicamycin, induced autophagy and apoptosis in MM cells, thereby inhibiting proliferation and chemotherapy resistance [35]. As a whole, the death of MM cells after 5,6-ECs treatment proceeds through oxidative stress, apoptotic and autophagic cell death mechanisms.

Finally, our data showed that the combination of low concentrations (IC_25_) of the two 5,6 α/β-ECs isomers increased the efficiency of the treatment in a synergistic manner (Table 2), as well the combination with bortezomib (Table 3). The synergy is a benefit over an additive response. In addition to the objective of maximizing efficiency, this could also improve the sensitivity and selectivity of cancer treatment. In MM therapy, synergistic drug combinations are highly sought after due to their multiple advantages, such as optimizing efficacy, reducing toxicity, and delaying or overcoming resistance. Accordingly, it was reported that therapeutic approaches based on the combination of two or more compounds would be more effective by affecting more signaling pathways and overcoming drug resistance mechanism [36]. In the literature, there are several examples of anticancer combination therapy using potent molecules [37].

5,6-ECs have been described as active biomolecules implicated in the pharmacology and/or therapeutic effects of anti-tumor drugs. In breast cancer cells, the isomers contribute to the anticancer pharmacology of tamoxifen through the inhibition of the enzymatic activity of cholesterol epoxide hydrolase resulting in the accumulation of endogenous 5,6 α/β-ECs [12,38]. We observed the same response in two HMCLs, where antiestrogen-binding site ligands induced apoptosis and autophagy through a mechanism that involve the production and the accumulation of the two isomers 5,6 α/β-ECs [13].

Investigating lipid metabolic pathway is a novel area in MM with preclinical data suggesting efficacy. Abnormal lipid and lipoprotein profiles have clearly been associated with MM. The level of total cholesterol, LDL-C and HDL-C were significantly lower in MM stages II and III patients than in controls [39]. Moreover, oxysterols intracellular concentration is correlated to that of cholesterol, and play an active role in cell signaling and cholesterol homeostasis [40]. Therefore, targeting cholesterol metabolism via epoxycholesterols in MM could be a potential approach to enhance anti-myeloma-based therapies.

## 5. Conclusions

To conclude, the present study provides evidence for a cytotoxic effect of 5,6-ECs α and β isomers on human myeloma cells. This anti-myeloma activity is concomitantly induced by oxidative stress, apoptosis and autophagy, a complex mode of cell death defined as oxiapoptophagy. The term oxiapoptophagy (OXIdative stress + APOPTOsis + autoPHAGY) firstly reported by Monier and coworkers [41], was later described to be induced by other oxysterols in multiple cell types [9]. Our study is the first to confirm the occurrence of oxiapoptophagy induced by 5,6-ECs in MM cells.

## Figures and Tables

**Table 1:** IC_50_ values of 5,6 α/β-EC for the different periods of treatment.

		24 h	48 h	72 h
JJN3	5,6 α-EC 5,6 β-EC	20 ± 2.2 31 ± 1.1	11 ± 0.4 14 ± 0.3	14 ± 0.5 7 ± 0.4
U266	5,6 α-EC 5,6 β-EC	26 ± 1.7 25 ± 0.5	31 ± 1.2 21 ± 0.4	30 ±0.8 12 ± 0.2

MM cell lines were seeded in 96-well plates at the density of 2 × 10^4^ cells per well, then treated for 24–72 h with vehicle, 5,6 α-EC or 5,6 β-EC (5–80 µg/mL). Cell viability was assessed using an MTT assay. The IC_50_ were calculated after drawing the inhibition curves using the Excel software and verified with the CompuSyn software (www.combosyn.com/, accessed on 21 July 2021). The indicated values are means ± SD from three different assays, with triplicate samples for each culture condition.

**Table 2:** Chou–Talalay index for the various combinations of 5,6-ECs.

5,6 α-EC (µg/mL)	5,6 β-EC (µg/mL)	CI-24 h	CI-48 h	Effect
12 *	20 *	0.18 ± 0.03	0.22 ± 0.2	Synergistic
26 **	27 **	8.52^−4^ ± 0.13	9.09^−4^ ± 0.24	Synergistic

U266 cell lines were seeded in 24-well plates at the density of 2 × 10^5^ cells per well, then treated for 24 h or 48 h the with the indicated concentrations of 5,6 α-EC or 5,6 β-EC corresponding to the IC_25_ * and IC_50_ **. Cell viability was assessed using an MTT assay. The CI, presented as means ± SD, were calculated with the CompuSyn software (www.combosyn.com/, accessed on 21 July 2021).

**Table 3:** Synergistic effect of 5,6-ECs associated with BTZ.

Cell	BTZ [nM]	5,6 α-EC [µg/mL]	5,6 β-EC [µg/mL]	CI (Mean ± SD)	Effect
**U266**	2	10	–	0.35 ± 0.087	Synergistic
	2	20	–	0.60 ± 0.024	Synergistic
	4	5	–	0.16 ± 0.037	Synergistic
	4	10	–	0.31 ± 0.039	Synergistic
	4	20	–	0.59 ± 0.014	Synergistic
	6	5	–	0.16 ± 0.019	Synergistic
	6	10	–	0.31 ± 0.015	Synergistic
	6	20	–	0.61 ± 0.012	Synergistic
	2	–	5	0.12 ± 0.02	Synergistic
	2	–	10	0.24 ± 0.02	Synergistic
	2	–	20	0.41 ± 0.02	Synergistic
	4	–	5	0.12 ± 0.01	Synergistic
	4	–	10	0.23 ± 0.01	Synergistic
	4	–	20	0.51 ± 0.01	Synergistic
	6	–	5	0.11 ± 0.02	Synergistic
	6	–	10	0.23 ± 0.03	Synergistic
	6	–	20	0.2 ± 0.21	Synergistic
**JJN3**	1.25	15	–	0.84 ± 0.05	Synergistic
	1.25	30	–	0.81 ± 0.02	Synergistic
	2.5	30	–	0.34 ± 0.01	Synergistic
	5	15	–	0.07 ± 0.04	Synergistic
	5	30	–	0.001 ± 0.01	Synergistic
	2	–	5	0.034 ± 0.05	Synergistic
	2	–	10	0.035 ± 0.01	Synergistic
	2	–	20	0.001 ± 0.02	Synergistic
	4	–	5	0.05 ± 0.01	Synergistic
	4	–	10	0.049 ±0.01	Synergistic
	4	–	20	0.001 ± 0.01	Synergistic
	6	–	5	0.05 ± 0.02	Synergistic
	6	–	10	0.50 ± 0.03	Synergistic
	6	–	20	$2.7^{-13}$ ± 0.21	Synergistic

MM cell lines were seeded in 96-well plates at the density of 5 × 10^4^ cells per well, then treated with the vehicle or 5,6 α-EC (5–30 µg/mL), 5,6 β-EC (5–20 µg/mL), BTZ (1.25–6 nM) alone or with 5,6 α-EC /BTZ, 5,6 β-EC/BTZ combination. Viability was revealed with an MTT assay after 48 h. The results were analyzed by the CompuSyn software to evaluate the combination index. The Chou–Talalay index offers a quantitative definition for additive effects (CI = 1.0), synergism (CI < 1.0), and antagonism (CI > 1.0) for drugs combination.

## Data Availability

The data presented in this study are available in the main text and supplementary materials.

## Supplementary Materials

The following are available online at https://www.mdpi.com/article/10.3390/cancers13153747/s1, Figure S1: Viability curves of HMCLs to 5,6 α/β-EC; Figure S2: 5,6 α-EC and 5,6 β-EC compounds lead to a time- and concentration-dependent loss of viability of JJN3 and U266 MM cells; Figure S3: 5,6 α-EC and 5,6 β-EC compounds exhibited anti-proliferative effects on JJN3 and U266 MM cells; Figure S4: The apoptosis triggered by 5,6 α/β-EC compounds leads to activation of caspase 3 and PARP cleavage; Figure S5: ROS-mediated cell death induced by the 5,6 α/β-EC compounds is partly reversed by vitamin E; Figure S6: ROS-mediated apoptosis is partly reversed by NAC in U266 cells; Figure S7: Autophagy participates in the cell death induced by the 5,6 α/β-EC compounds; Figure S8: 5,6 α-EC and 5,6 β-EC isomers act synergistically on MM cells; Table S1: Characteristics of the HMCLs used in the study; Table S2: Clinico-biological parameters of MM patients; Table S3: Statistical analyses of Figure 2A; Table S4: Annexin V/PI assay results; Table S5: Statistical analyses of Figure 2B,D; Table S6: Statistical analyses of Figure 3A; Table S7: Statistical analyses of Figure 3B,C; Supplementary references.
