# Supplementary material for: 5,6-Epoxycholesterol Isomers Induce Oxiapoptophagy in Myeloma Cells
Source: Cancers (Basel). 2021 Jul 26;13(15):3747. doi: 10.3390/cancers13153747 (PMC8345143; doi:10.3390/cancers13153747)
Supplement: Supplementary file 1 [file cancers-13-03747-s001.zip › cancers-1282184-supplementary.pdf]

cancers

MDPI

Supplementary

5,6 epoxycholesterol isomers induce oxiapoptophagy in myeloma cells

Oumaima Jaouadi, Inès Limam, Mohamed Abdelkarim, Emna Berred, Ahlem Chahbi, Mélody Caillot, Brigitte Sola and Fatma Ben Aissa-Fennira

1. Supplementary methods

1.1. FDA viability assay

The fluorescein diacetate (FDA) assay was performed to estimate the number of viable cells by quantification of FDA enzymatic cleavage in fluorescein [1]. JJN3 and U266 cells were seeded for 24 h at the density of 2 x 10^5 cells/well in 96-well black culture microplates. After 5-80 µg/mL 5,6 α-EC or 5,6βEC treatment for 24-72 h, cells were mixed with 10 µM FDA (#F7378, Sigma-Aldrich, Saint-Louis, MO) and incubated for 10 min at 37°C in the dark. The enzymatic cleavage of the FDA molecule (colorless) into fluorescein (green fluorescence) was detected and quantified by microplate reader (Victor X2, Perkin Elmer, Waltham, MA).

1.2. Effect of ROS production on MM cell apoptosis

U266 cells were seeded in 24-well plates (2 x 10^5 cells/well) for 24 h, pre-treated (or not for control) with 1 mM N-acetyl-L-cysteine (NAC, A7250, Sigma-Aldrich) then treated with 5,6 β-EC (20 or 40 µg/mL). After 24 h of treatment, cells were stained with annexin V-FITC/PI for apoptosis assessment. At least, 10^4 events per sample were acquired and analyzed by flow cytometry with the CytoFlex cytometer and the CytExpert software (Beckman Coulter, Pasadena, CA).

2. Supplementary Figures

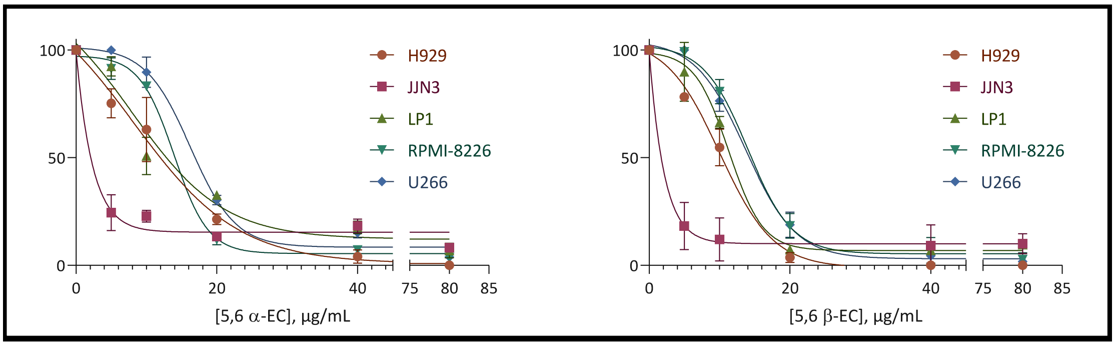

Figure S1. Viability curves of HCMLs to 5,6 α-EC and 5,6β-EC. The indicated five MM cell lines were assayed for their sensitivity towards both 5,6-EC isomers. Cells were seeded in 96-well plates at a density of 5 x 10^4 cells/well and treated with vehicle or increasing concentrations of drugs (5–80 µg/mL) for 48 h. The viability of each cell line treated with the drug, determined by an MTT assay, is expressed relative to that of the cell line treated with the vehicle (EtOH, defined as 100%). For each culture condition, the mean of triplicate ratios is indicated on the graph, together with the SD. The curves have been drawn with the Prism software (v8.0).

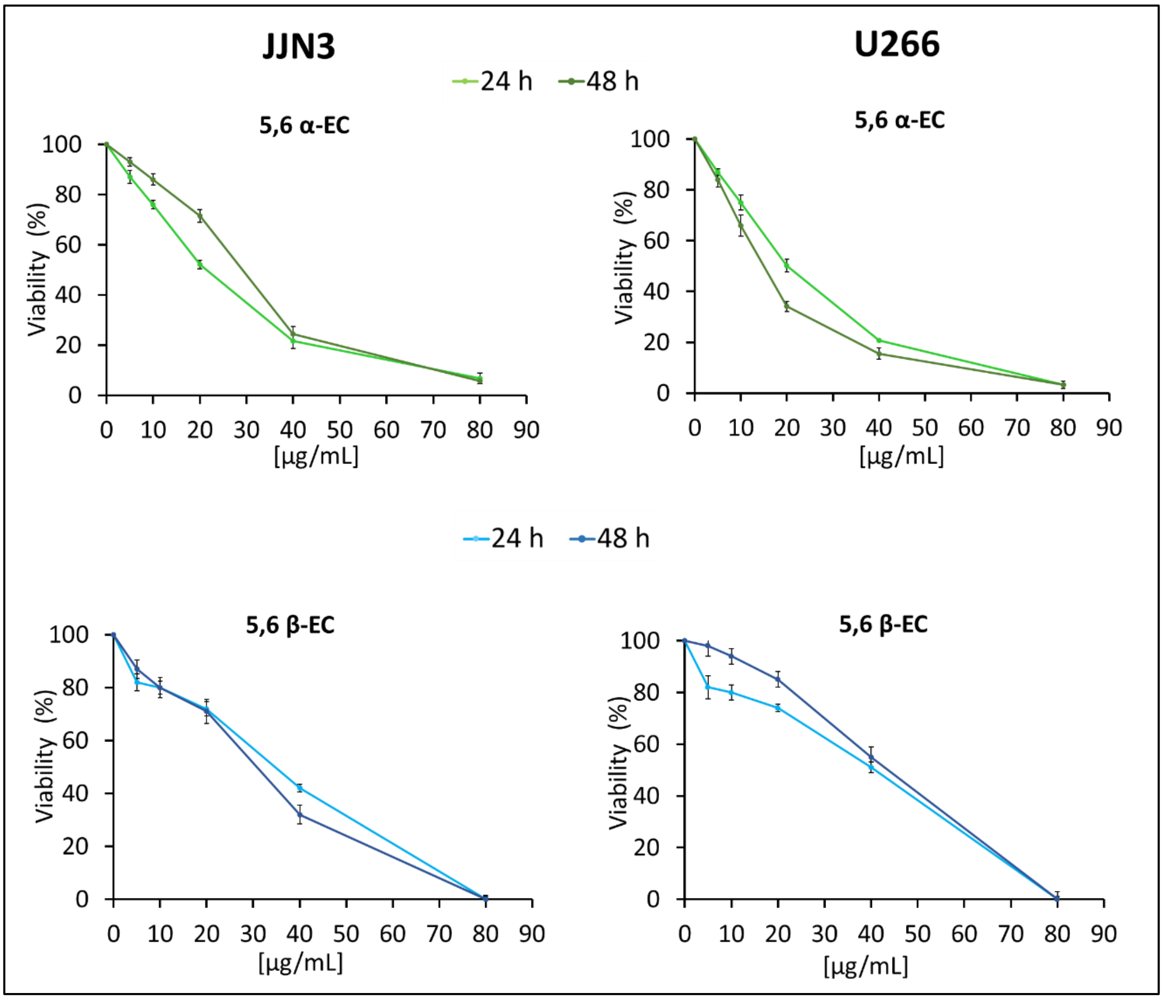

Figure S2. 5,6 α-EC and 5,6 β-EC compounds lead to a time- and concentration-dependent loss of viability of JJN3 and U266 MM cells. JJN3 and U266 cells were seeded in 96-well black microplates, then cultured in the absence or the presence of 5,6 α-EC or 5,6 β-EC used at 5-80 µg/mL for 24-72 h. Viability assays were performed using the colorimetric FDA fluorometric assay. Values are expressed as means ± SD of 3 different experiments. No statistically significant difference between control and vehicle (EtOH)) was noticed.

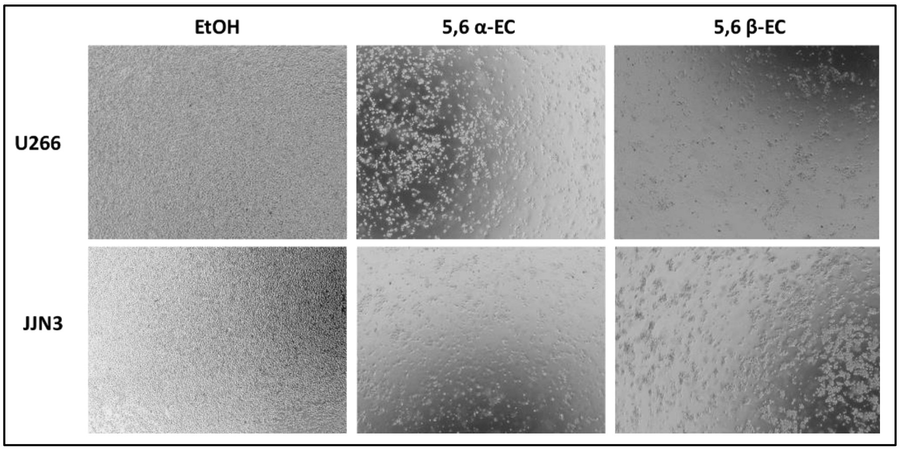

Figure S3. 5,6 α-EC and 5,6 β-EC compounds exhibited anti-proliferative effects on JJN3 and U266 MM cells. JN3 and U266 cells were seeded in 24-well plates for 24 h at the density of 2 x 10^5 cells per well and treated for 48 h with 40 µg/mL 5,6 α-EC or 5,6 β-EC or EtOH. Cell morphology and density were visualized by a phase-contrast inverted. microscope (LEICA DM1000, Leica microsystems, Germany), images were obtained with a digital camera (×200, magnification).

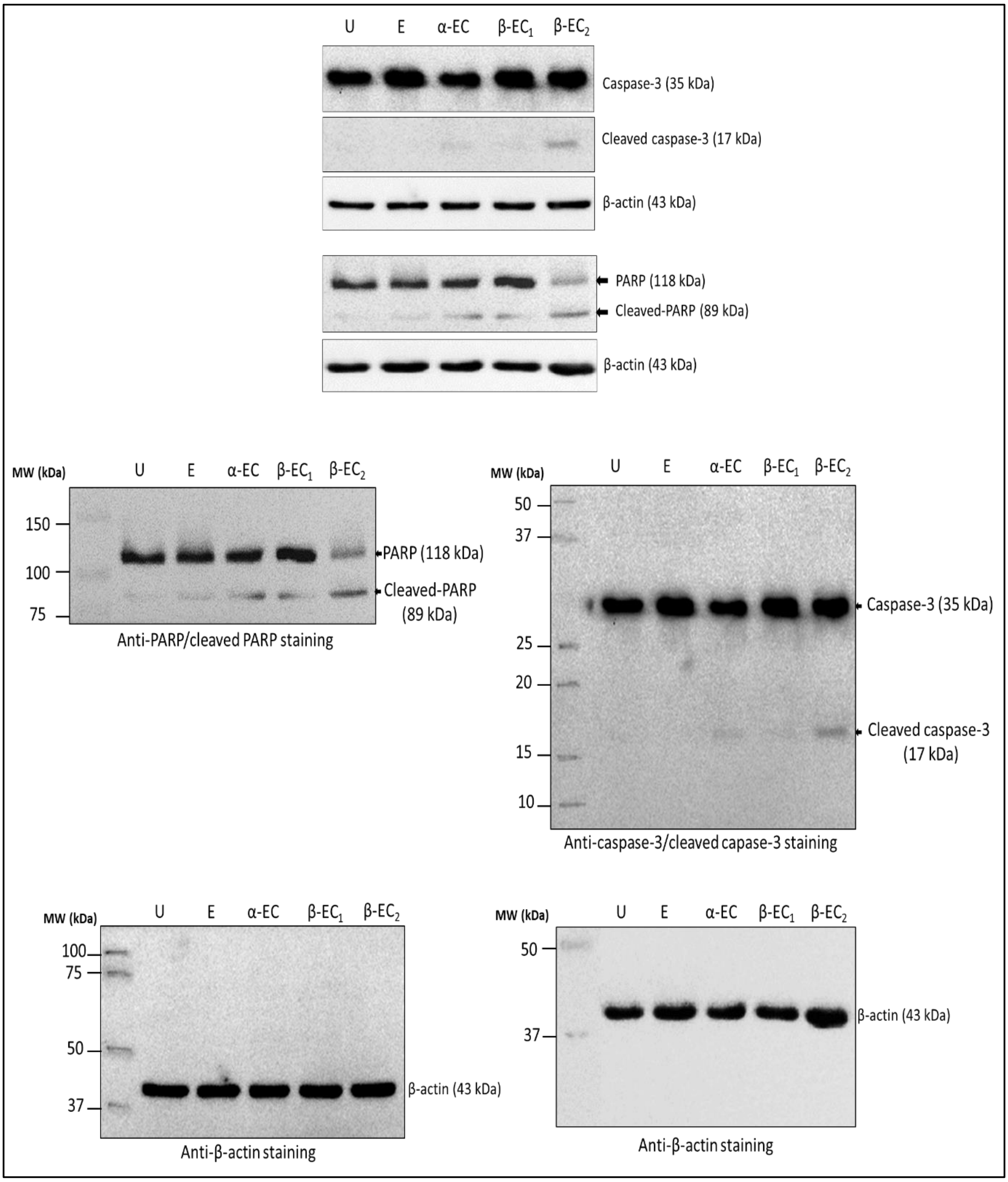

Figure S4. The apoptosis induced by 5,6 α/β-EC necessitates the activation of the caspase 3 executioner caspases and lead to the cleavage of PARP. The activation of caspase 3 (cleaved) and the cleavage of poly(ADP) ribose polymerase (PARP) were evaluated by western blotting. U266 cells were untreated (U), vehicle-treated (E) or treated with 5,6 α-EC (40 μg/mL) or 5,6 β-EC (20 or 40 μg/mL, β-EC_1 or β-EC_2, respectively). Within 24 h, whole-cell proteins were extracted, separated by SDS-PAGE, and transferred onto membranes then incubated with anti-caspase 3 (#9662, 1/1,000 dilution), anti-PARP (#9532, 1/1,000 dilution) or anti-β-actin (#4970, 1/10,000 dilution) antibodies all provided by Cell Signaling Technologies (Danvers, MA). The anti-β-actin antibody was used as a control of loading and transfer. Original blots are presented.

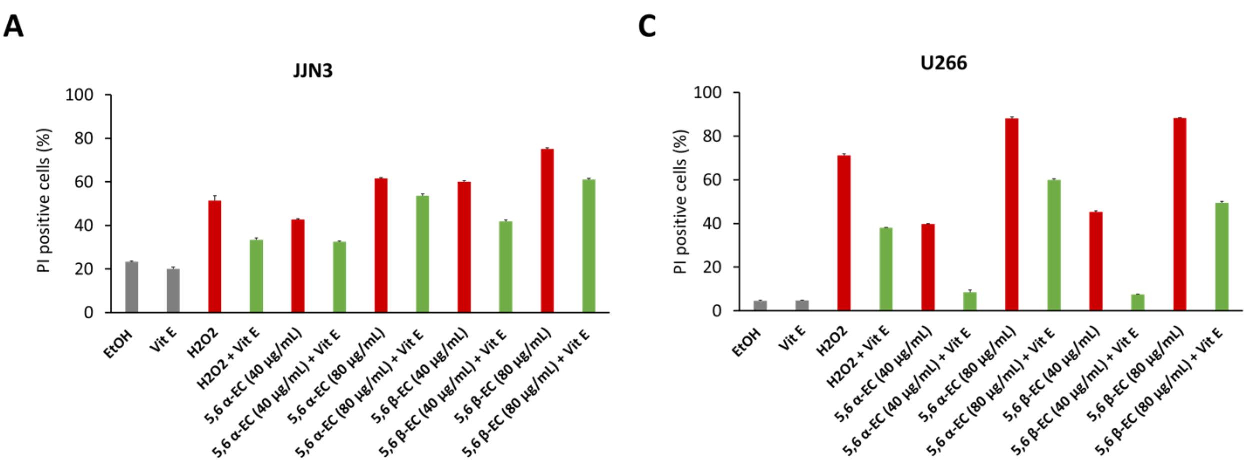

B

JJN3		
**Treatment 1**	**Treatment 2**	**p value**
H_2O_2	EtOH	<0,001
H_2O_2 + Vit E	H_2O_2	<0,001
5,6 α-EC (40 µg/mL)	EtOH	<0,001
5,6 α-EC (40 µg/mL) + Vit E	5,6 α-EC (40 µg/mL)	<0,001
5,6 α-EC (80 µg/mL)	EtOH	<0,001
5,6 α-EC (80 µg/mL) + Vit E	5,6 α-EC (80 µg/mL)	0,006
5,6 β-EC (40 µg/mL)	EtOH	<0,001
5,6 β-EC (40 µg/mL) + Vit E	5,6 β-EC (40 µg/mL)	<0,001
5,6 β-EC (80 µg/mL)	EtOH	<0,001
5,6 β-EC (80 µg/mL) + Vit E	5,6 β-EC (80 µg/mL)	<0,001

D

U266		
**Treatment 1**	**Treatment 2**	**p value**
H_2O_2	ETOH	<0,001
H_2O_2 + Vit E	H_2O_2	<0,001
5,6 α-EC (40 µg/mL)	ETOH	<0,001
5,6 α-EC (40 µg/mL) + Vit E	5,6 α-EC (40 µg/mL)	<0,001
5,6 α-EC (80 µg/mL)	ETOH	<0,001
5,6 α-EC (80 µg/mL) + Vit E	5,6 α-EC (80 µg/mL)	<0,001
5,6 β-EC (40 µg/mL)	ETOH	<0,001
5,6 β-EC (40 µg/mL) + Vit E	5,6 β-EC (40 µg/mL)	<0,001
5,6 β-EC (80 µg/mL)	ETOH	<0,001
5,6 β-EC (80 µg/mL) + Vit E	5,6 β-EC (80 µg/mL)	<0,001

Figure S5. ROS-mediated cell death induced by the 5,6 α/β-EC compounds is partly reversed by vitamin E**.** The inhibition of cell death induced by 5,6α/β-ECs-mediated oxidative stress was evaluated by the PI staining of JJN3 cells (**A**) and U266 cells (**B**). Cells were pretreated (or not, for a control) with 400 µM vitamin E (Vit E) for 2 h, then treated with 5,6 α-EC or 5,6 β-EC (40-80 µg/mL) for 48 h. The percentages of PI+ cells are presented as the means ± SD from three independent experiments done with triplicate samples. Statistical *t*-test was used to calculate *p*-values for JJN3 (**B**) and U266 (**D**) treated cells between 5,6-EC vs vehicle and 5,6-EC vs 5,6-EC+Vit E. No statistically significant difference between control and EtOH was noticed.

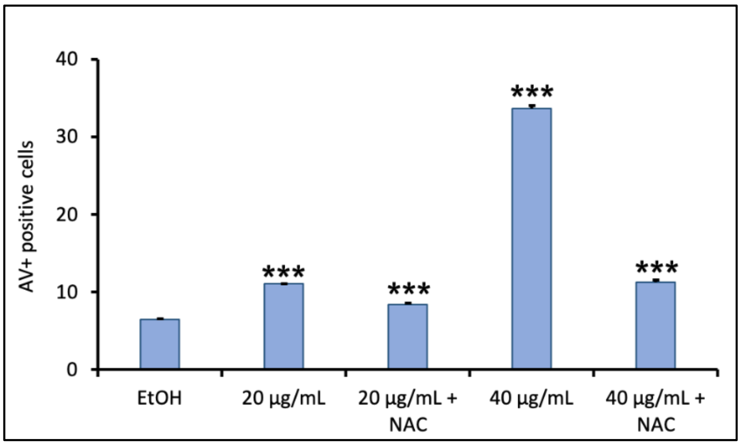

Figure S6. ROS-mediated apoptosis induced by the 5,6 β-EC compound is partly reversed by NAC in U266 cells. U266 cells were cultured in 24-well plates (5 x 10^5 cells/well), pretreated or not with NAC (1 mM) for 12 h, then, treated for 24 h with 5,6 β-EC (20 or 40 µg/mL). The inhibition of apoptosis induced by 5,6 β-EC was evaluated by the percentage of AV+ cells. Means ± SD are presented in the histograms**.** Statistical *t*-tests were used to calculate the *p*-values between 5,6 β-ECs-treated cells vs. vehicle-treated cells and between 5,6β-EC- vs. 5,6β-ECs + NAC-treated cells *** $p < 0.001$. No statistically significant difference between control and vehicle (EtOH) was noticed.

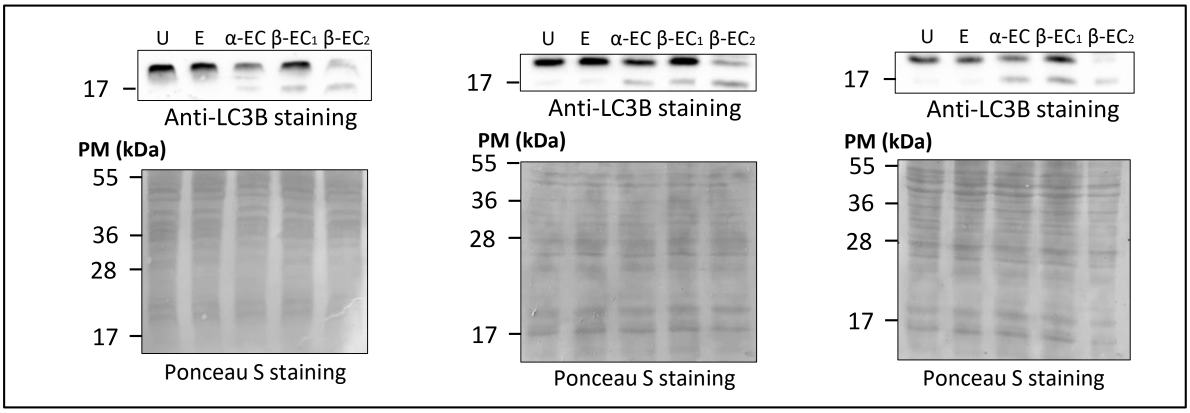

Figure S7. Autophagy participates in the cell death induced by the 5,6 α/β-EC compounds. U266 cells were cultured in the presence of 5,6 α-EC (40 µg/mL) or 5,6 β-EC (20 or 40 µg/mL, β-EC1 or β-EC2, respectively) for 24 h. Whole-cell protein were extracted, separated by SDS-PAGE, and transferred onto membranes then incubated with an anti-LC3B antibody (#52520, abcam, 1/1,000 dilution). Protein levels were estimated by densitometry from three independent experiments. The corresponding original blots are presented.

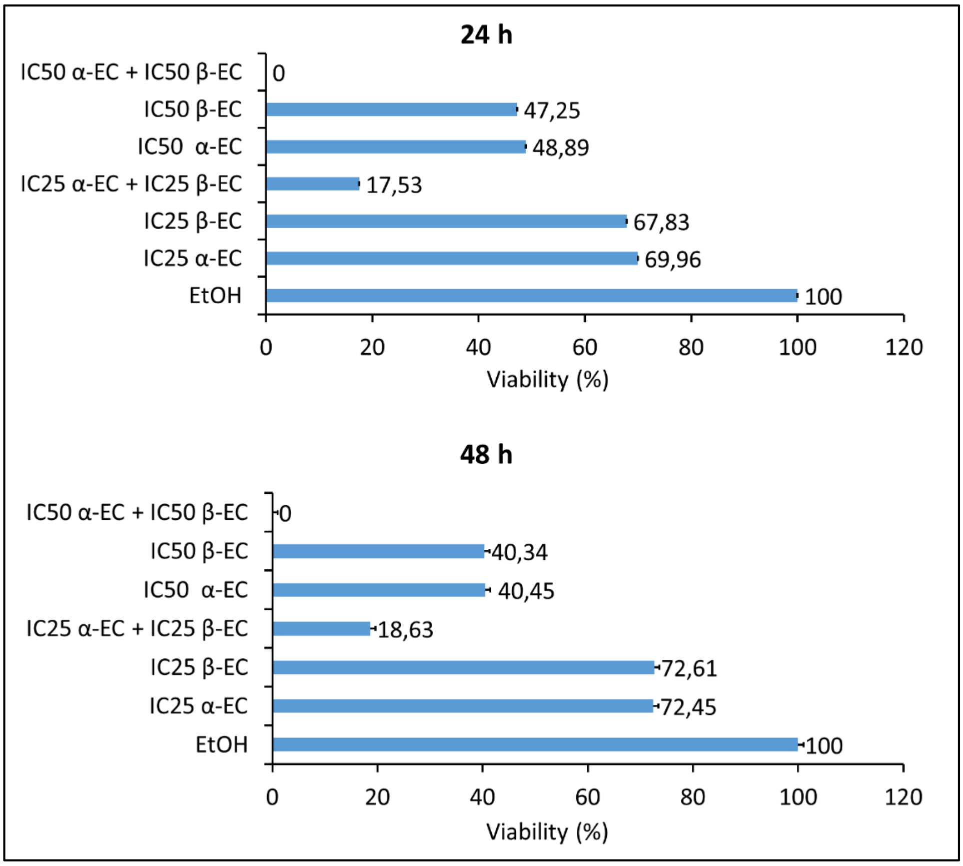

Figure S8. 5,6 α-EC and 5,6 β-EC isomers act synergistically on MM cells. The synergistic effects of 5,6 α-EC and 5,6 β-EC isomers were investigated by the MTT assay. U266 cells were seeded in 96-well plates for 24 h at the density of 2 × 10^4 cells/well then treated for 24 or 48 h with the corresponding IC_{25} or IC_{50} concentrations. Cells were either treated with IC_{25} 5,6 α-EC (12 or 21 µg/mL), IC_{25} 5,6 β-EC (20 or 12 µg/mL), IC_{50} 5,6 α-EC (26 or 31 µg/mL), IC_{50} 5,6 β-EC (27 or 21 µg/mL) separately or with the combination of 5,6 α/β-EC IC_{25} or 5,6 α/ β-EC IC_{50} for 24 h and 48 h respectively. Cell viability in these various culture conditions was evaluated by MTT assay as described previously. Percentages of viability are expressed as means ± SD in the graph. Combination index (CI) calculated by Chou-Talalay method using the CompuSyn software are in the Table 2.

3. Supplementary Tables

Table S1. Characteristics of the HMCLs used in the study.

Name	Disease	Sample	Gender/age	Isotype	Translocation	Group	BTZ IC_{50} (nM)
JJN3	MM	PE	F/57	A/κ	t(14;16)	MF	4.6
LP1	MM	PB	F/56	G/λ	t(4;14)	MS	9.8
18%NCI-H929	MM	PE	F/62	A/κ	t(4;14)	MS	3.7
RPMI 8226	MM	PB	M/61	λ	t(14;16)	MF	9.1
U266	MM	PB	M/53	E/λ	t(11;14)	CD-1	4.8

Abbreviations: BTZ, bortezomib; MM, multiple myeloma; nd, not done; PB, peripheral blood; PCL, plasma cell leukemia; PE, pleural effusion. The group was defined according to the molecular classification of Zhan *et al.* [2]. The table was adapted from Moreaux *et al.* [3]. The IC_{50} towards BTZ have been described previously [4].

Table S2. Clinico-biological parameters of MM patients.

Patient	Infiltration	Gender	Age (y)	IgH/L	ISS	Symptoms	Chromosome abnormality	Treatment response
P1	18%	M	55	IgG/κ	stage IIIA	anemia	no	91% after 4 VTD
P2	45%	M	39	IgA/κ	stage IIIC	anemia, hypercalcemia, inugural spinal cord compression	t(4;14)	96% after 4 VTD
P3	29%	F	50	IgG/κ	stage IIIC	bone lesions, compressive plasmacytoma	no	95% after 4 VTD
P4	20%	M	56	IgG/κ	stage IIIA	hypercalcemia	del(17p)	86% after 4 VTD
P5	13%	M	51	IgG/κ	nd	anemia	nd	Long-lost patient

Abbreviations: ISS, international staging system; nd, not done; VTD, velcade, thalidomide, dexamethasone.

Table s3. Statistical analyses of Figure 2A.

	5,6 α-EC [µg/mL]			5,6 β-EC [µg/mL]		
	24 h	48 h	72 h	24 h	48 h	72 h
JJN3						
EtOH vs 20	0.055	0.011	<0.001	<0.001	0.0075	0.0056
EtOH vs 40	0.012	<0.001	<0.001	<0.001	0.0420	0.0014
EtOH vs 80	<0.001	0.0073	<0.001	ns	0.0920	<0.001
U266						
EtOH vs 20	ns	ns	0.024	0.0072	0.046	<0.001
EtOH vs 40	0.0067	0.049	0.01	<0.001	0.0031	<0.001
EtOH vs 80	0.0094	0.0091	0.058	<0.001	0.0036	0.0016

JJN3 and U266 cell lines were seeded in 24-well plates at a density of $2x10^4$ cells per well for 24 h, then treated for 24-72 h with vehicle, 5,6α-EC or 5,6β-EC (20-80 µg/mL). The percentage of cells in sub-G1 cell cycle phase was determined after PI staining using flow cytometry. Statistical *t*-test was used to calculate the *p*-values presented in the table for the comparison between the different culture conditions.

Table S4. Annexin V/PI assay results.

	5.6α-EC [μg/mL]			5.6β-EC [μg/mL]		
	Live Cells	Apoptotic Cells	Necrotic Cells	Live Cells	Apoptotic Cells	Necrotic Cells
**JJN3**						
24 h						
EtOH	80	18.1	1.9	93.2	6.4	0.5
20	76.2	18.6	5.2	77.6	14.6	7.8
40	72.7	20.6	6.6	11	37.0	52.1
80	15.4	61.1	23.4	2.9	19.6	77.6
48 h						
EtOH	87.2	10.8	2	92.8	5.6	1.5
20	53.6	39.5	6.9	28.7	4	67.3
40	9.5	32.1	58.5	9.6	4.4	86.2
80	5.5	20.6	73.6	6.7	5.8	87.6
72 h						
EtOH	90.6	8.4	1	94.2	5.3	0.5
20	27.6	52	20.2	10.5	3.4	86.1
40	3.4	55	41.3	3.4	6.8	89.8
80	5	34.1	60.8	2.5	6	91.3
**U266**						
24 h						
EtOH	91.5	7.8	0.7	94.2	5.7	0.1
20	91.6	5.3	2.7	65	32.9	2
40	88.2	6.6	3.8	11.8	77.7	10.6
80	84.8	6.5	7.8	9.8	82.4	7.8
48 h						
EtOH	95.7	3.9	0.4	92.6	7.1	0.2
20	92.6	5.1	2.2	36.7	46.3	16.9
40	55.6	33.6	10.8	9.1	71.3	19.6
80	4.3	48.0	47.8	5.6	10.1	84.3
72 h						
EtOH	93.8	6	0.2	93.1	6.3	0.5
20	92.6	6.4	0.7	11.2	71.4	17.3
40	29.9	63.3	6.9	7.5	81.5	11
80	17.2	40.1	42.7	2.6	3.3	94.1

MM cell lines were seeded in 24-well plates at the density of 2×10^4 cells per well for 24 h, then treated for 24-72 h with vehicle, 5,6 α-EC or 5,6 β-EC (20-80 μg/mL). The percentage of cells in each compartment (living cells, apoptotic cells (early and late apoptosis) or necrotic cells was determined after staining with annexin V /PI assay and sorting by flow cytometry.

Table S5. Statistical analyses of Figure 2B-D.

Cell lines	5,6α-EC [μg/mL]				5,6β-EC [μg/mL]			
	AV-/PI-	AV+/PI-	AV+/PI+	AV-/PI+	AV-/PI-	AV+/PI-	AV+/PI+	AV-/PI+
**JJN3**								
24 h								
EtOH vs 20	ns	ns	ns	ns	0.017	0.027	<0.001	0.0095
EtOH vs 40	0.005	ns	0.039	0.023	<0.001	0.0054	0.0069	0.0036
EtOH vs 80	<0.001	0.0058	<0.001	<0.001	<0.001	0.0036	0.0016	<0.001
48 h								

EtOH vs 20	<0.001	0.0046	0.0077	ns	0.0052	0.031	ns	<0.001
EtOH vs 40	<0.001	ns	0.011	0.0054	<0.001	0.022	ns	<0.001
EtOH vs 80	<0.001	0.0039	<0.001	<0.001	<0.001	0.024	ns	<0.001
72 h								
EtOH vs 20	<0.001	0.018	0.0072	<0.001	<0.001	<0.001	ns	<0.001
EtOH vs 40	<0.001	0.0098	0.0052	0.01	<0.001	<0.001	ns	<0.001
EtOH vs 80	<0.001	0.015	<0.001	<0.001	<0.001	<0.001	ns	<0.001
**U266**								
24 h								
EtOH vs 20	ns	ns	ns	ns	<0.001	0.0071	<0.001	0.012
EtOH vs 40	ns	ns	ns	0.001	<0.001	0.0011	<0.001	<0.001
EtOH vs 80	ns	0.047	ns	<0.001	<0.001	<0.001	<0.001	0.015
48 h								
EtOH vs 20	0.048	ns	ns	ns	0.0011	<0.001	<0.001	0.0042
EtOH vs 40	<0.001	0.001	<0.001	0.001	<0.001	0.024	0.003	0.03
EtOH vs 80	<0.001	0.011	<0.001	<0.001	<0.001	0.0032	0.049	<0.001
72 h								
EtOH vs 20	ns	ns	ns	ns	<0.001	0.0083	0.0027	0.0093
EtOH vs 40	<0.001	0.0039	<0.001	<0.001	<0.001	<0.001	0.0012	0.019
EtOH vs 80	<0.001	ns	<0.001	<0.001	<0.001	<0.001	ns	<0.001

MM cell lines were seeded in 24-well plates at the density of $2x10^4$ cells per well for 24 h , then treated for 24-72 h with vehicle, 5,6α-EC or 5,6β-EC (20-80 μg/mL). The percentage of cells in each compartment (live cells, early apoptosis, late apoptosis or necrosis) was determined with an annexin V (AV)/propidium iodide (PI) assay performed using flow cytometry. The Student *t*-test was used to calculate *p*-values for the comparison between the vehicle culture condition vs drug condition. The calculated values are reported in the Table. ns, not significant.

Table 6. Statistical analyses of Figure 3A.

**Cell lines**	**5,6α-EC [μg/mL]**			**5,6β-EC [μg/mL]**		
	24 h	48 h	72 h	24 h	48 h	72 h
**JJN3**						
EtOH vs 20	0.011	0.0017	<0.001	0.0072	0.046	<0.001
EtOH vs 40	0.0067	<0.001	<0.001	<0.001	<0.001	<0.001
EtOH vs 80	<0.001	<0.001	<0.001	<0.001	<0.001	<0.001
EtOH vs H_2O_2	<0.001	<0.001	<0.001	<0.001	<0.001	0.0032
**U266**						
EtOH vs 20	ns	ns	ns	<0.001	<0.001	<0.001
EtOH vs 40	0.0095	<0.001	<0.001	<0.001	<0.001	<0.001
EtOH vs 80	<0.001	<0.001	<0.001	<0.001	<0.001	<0.001
EtOH vs H_2O_2	0.0037	<0.001	<0.001	<0.001	<0.001	<0.001

U266 and JJN3 cell lines were seeded in 24-well plates at a density of $2x10^4$ cells per well for 24 h, then treated for 24-72 h with vehicle, 5,6α-EC, 5,6β-EC (20-80 μg/mL) or H_2O_2 (500 μM) as a control. The percentage of percentage of cells with activated caspase3/7 was determined by flow cytometry with the CellEvent caspase 3/7 *G*reen Flow *C*ytometry assay kit (Molecular Probes) according to the manufacturer' instructions. At least 10^4 events were gated for each condition. The Student *t*-test was used to calculate the *p*-values presented in the table for the comparison between the different culture conditions. ns, not significant.

Table S7. Statistical analyses of Figure 3B,C.

**Cell lines**	**5,6α-EC [μg/mL]**			**5,6β-EC [μg/mL]**		
	24 h	48 h	72 h	24 h	48 h	72 h
JJN3						
EtOH vs 20	<0.001	<0.001	<0.001	0.0096	0.001	<0.001
EtOH vs 40	0.0019	<0.001	<0.001	<0.001	<0.001	<0.001
EtOH vs 80	<0.001	<0.001	<0.001	<0.001	<0.001	<0.001
EtOH vs H_2O_2	<0.001	0.047	<0.001	0.0014	0.0015	<0.001
U266						
EtOH vs 20	0.027	0.0044	0.044	0.0039	<0.001	0.001
EtOH vs 40	0.012	<0.001	<0.001	<0.001	<0.001	0,0015
EtOH vs 80	0.016	<0.001	0.0021	<0.001	<0.001	<0.001
EtOH vs H_2O_2	0.0037	<0.001	<0.001	<0.001	<0.001	<0.001

U266 and JJN3 cell lines were seeded in 24-well plates at a density of $2x10^4$ cells per well for 24 h, then treated for 24-72 h with vehicle, 5,6α-EC, 5,6β-EC (20-80 μg/mL) or H_2O_2 (500 μM) as a control. The percentage of $DIOC_6$-(3) negative cells was recorded according to the manufacturer' instructions. At least 10^4 events were gated for each condition. The Student *t*-test was used to calculate the *p*-values presented in the table for the comparison between the different culture conditions.

Table S8. Statistical analyses for Figure 4B.

Treatment 1	Treatment 2	JJN3	U266
H_2O_2	EtOH	<0.001	<0.001
H_2O_2 + VitE	H_2O_2	<0.001	<0.001
5,6 α-EC (40 μg/mL)	EtOH	0.018	<0.001
5,6 α-EC (40) +VitE	5,6 α-EC (40 μg/mL)	<0.001	<0.001
5,6 α-EC (80 μg/mL)	EtOH	<0.001	<0.001
5,6 α-EC (80) +VitE	5,6 α-EC (80 μg/mL)	<0.001	<0.001
5,6 β-EC (40 μg/mL)	EtOH	<0.001	<0.001
5,6 β-EC (40) +VitE	5,6 β-EC (40 μg/mL)	<0.001	<0.001
5,6 β-EC (80 μg/mL)	EtOH	<0.001	<0.001
5,6 β-EC (80) +VitE	5,6 β-EC (80 μg/mL)	<0.001	<0.001

JJN3 and U266 cells were treated or not with 400 μM Vit E for 2 h then, treated for 24 h with 5,6 α-EC or 5,6 β-EC (40-80 μg/mL). The inhibition of death induced was evaluated by the percentage of PI+ HMCLs as described. The calculated *p*-values between treatment 1 and 2 are presented in the Table.

4. Supplementary references

1. Vitecek, J.; Petrlova, J.; Adam, V.; Havel, L.; Kramer, K.; Babula, P.; Kizek, R. A Fluorimetric sensor for detection of one living cell. *Sensors* **2007**, *7*, 222–238, doi:10.3390/s7030222.
2. Zhan, F.; Huang, Y.; Colla, S.; Stewart, J.P.; Hanamura, I.; Gupta, S.; Epstein, J.; Yaccoby, S.; Sawyer, J.; Burington, B.; Anaissie, E.; Hollmig, K.; Pineda-Roman, M.; Tricot, G.; van Rhee, F.; Walker, R.; Zangari, M.; Crowley, J.; Barlogie, B.; Shaughnessy, J.D.Jr. The molecular classification of multiple myeloma. *Blood* **2006**, 108, 2020-2028, doi: 10.1182/blood-2005-11-013458.
3. Moreaux, J.; Klein, B.; Bataille, R.; Descamps, G.; Maïga, S.; Hose, D.; Goldschmidt, H.; Jauch, A.; Rème, T.; Jourdan, M.; Amiot, M.; Pellat-Deceunynck, C. A high-risk signature for patients with multiple myeloma established from the molecular classification of human myeloma cell lines. *Haematologica* **2011**, 96, 574-582, doi: 10.3324/haematol.2010.033456.
4. Caillot, M.; Zylbersztejn, F.; Maitre, E.; Bourgeais, J.; Hérault, O.; Sola, B. ROS overproduction sensitises myeloma cells to bortezomib-induced apoptosis and alleviates tumour microenvironment-mediated cell resistance. *Cells* **2020**, 9, 2357, doi: 10.3390/cells9112357
